# Increasing dietary levels of the *n*-3 long-chain PUFA, EPA and DHA, improves the growth, welfare, robustness and fillet quality of Atlantic salmon in sea cages

**DOI:** 10.1017/S0007114522000642

**Published:** 2023-01-14

**Authors:** Esmail Lutfi, Gerd M. Berge, Grete Bæverfjord, Trygve Sigholt, Marta Bou, Thomas Larsson, Turid Mørkøre, Øystein Evensen, Nini H. Sissener, Grethe Rosenlund, Lene Sveen, Tone-Kari Østbye, Bente Ruyter

**Affiliations:** 1 Nofima (Norwegian Institute of Food, Fisheries and Aquaculture Research), Ås N-1432, Norway; 2 Nofima, Sunndalsøra, Norway; 3 BioMar AS, Trondheim, Norway; 4 Department of Animal and Aquacultural Sciences, Norwegian University of Life Sciences, Ås, Norway; 5 Faculty of Veterinary Medicine, Norwegian University of Life Sciences, Oslo, Norway; 6 Institute of Marine Research, Bergen, Norway; 7 Skretting ARC, Stavanger, Norway

**Keywords:** EPA, DHA, Aquaculture, Fish nutrition

## Abstract

The present study evaluated the effects of increasing the dietary levels of EPA and DHA in Atlantic salmon (*Salmo salar*) reared in sea cages, in terms of growth performance, welfare, robustness and overall quality. Fish with an average starting weight of 275 g were fed one of four different diets containing 10, 13, 16 and 35 g/kg of EPA and DHA (designated as 1·0, 1·3, 1·6 and 3·5 % EPA and DHA) until they reached approximately 5 kg. The 3·5 % EPA and DHA diet showed a significantly beneficial effect on growth performance and fillet quality compared with all other diets, particularly the 1 % EPA and DHA diet. Fish fed the diet containing 3·5 % EPA and DHA showed 400–600 g higher final weights, improved internal organ health scores and external welfare indicators, better fillet quality in terms of higher visual colour score and lower occurrence of dark spots and higher EPA and DHA content in tissues at the end of the feeding trial. Moreover, fish fed the 3·5 % EPA and DHA diet showed lower mortality during a naturally occurring cardiomyopathy syndrome outbreak, although this did not reach statistical significance. Altogether, our findings emphasise the importance of dietary EPA and DHA to maintain good growth, robustness, welfare and fillet quality of Atlantic salmon reared in sea cages.

The aquaculture industry has grown rapidly over the past few decades and has become one of the globally most important food-producing sectors. It currently provides half of the fish consumed worldwide and is estimated to surpass wild catch for the first time within the next few years^(1)^. This rapid development, together with the subsequent intensification of aquafeed production, has led to an increase in fish nutrition research, which has focused on improving productivity while reducing the use of marine ingredients in fish diets. In this regard, efforts have been made to replace limited sources, such as fish meal (FM) and fish oil (FO), with products of plant origin^(2–4)^. In Norway, the use of FM and FO in Atlantic salmon (*Salmo salar*) diets has decreased from 90 % (65 % FM, 24 % FO) to less than 30 % (14 % FM, 10 % FO) in the last few decades^(5)^. While this shift has lowered the dietary reliance on finite marine-derived ingredients, it has also led to a significant reduction in the use of the nutritionally beneficial *n*-3 long-chain PUFA, EPA (20:5 *n*-3) and DHA (22:6 *n*-3) in Atlantic salmon tissues^(6,7)^. In addition, the concomitant high inclusion levels of some vegetable ingredients that are rich in *n*-6 PUFA, associated with increased risk of inflammatory diseases^(8)^, may result in a less desirable enhanced *n*-6/*n*-3 ratio in fish organs and tissues and therefore impact salmon’s nutritional quality.

Fish, like all vertebrates, have a modest capacity to synthesise *n*-3 PUFA *de novo*; therefore, these must be obtained through their diet. EPA and DHA have numerous physiological functions in the body, such as preservation of the structure and integrity of cellular membranes, regulation of several processes related to immunity and inflammation and modulation of carbohydrate and lipid metabolism^(9,10)^. In previous studies, the dietary requirements of the *n*-3 PUFA, EPA and DHA, and their precursor, *α*-linolenic acid (18:3 *n*-3), in salmonids were reported to range from 1 to 2·5 % of the feed^(11–13)^. Several studies have demonstrated that reduced dietary EPA and DHA levels have a detrimental impact on fish growth and survival. For instance, in salmon, reducing dietary EPA and DHA levels to 1 % in the diet or below decreased fish growth and significantly increased mortality in demanding environmental conditions compared with fish fed higher levels of these PUFA^(14,15)^. Moreover, recent studies have suggested that deficiencies of these essential fatty acids (FA) may also affect other aspects, such as fish welfare, robustness and flesh quality. For example, low dietary levels of EPA and DHA have been shown to increase the amount of fat in the liver and viscera, induce morphological abnormalities in the intestine^(14)^ and increase the presence of melanin spots in the fillet^(16)^. Even in the absence of detrimental symptoms or direct effects on growth, low levels of EPA and DHA can have an important economic impact on commercial long-term production in sea cages, where fish face numerous environmental and health-related challenges, such as temperature fluctuation and parasite and pathogen infections. Some studies have shown that the EPA and DHA levels in heart and muscle tissues of Atlantic salmon may influence the severity of inflammatory responses to viral and sea-lice infections^(17,18)^. Taken together, it is clear that defining the dietary requirements of these essential FA in salmon is crucial to ensure growth as well as optimal fish health and quality.

Recent studies have examined the beneficial effects of increasing dietary EPA and DHA levels in salmon diets with the aim to minimise the potential negative impact of replacing FM and FO with vegetable ingredients. About 1·6 % EPA and DHA (1:1 ratio) is regarded as safe and does not reduce growth or fish robustness^(14,16,19)^. However, little is known about the potential benefits of including more than 2·0 % EPA and DHA in the diet. Therefore, the present study aimed to reassess the potential beneficial effects of increased levels of EPA and DHA in salmon diets, focusing on growth, performance, welfare and health, as well as their influence on fillet quality and *n*-3 PUFA content in different organs and tissues. This feeding trial was conducted over more than 1 year in fish reared in sea cages to mimic the environmental conditions that salmon face in commercial-scale production farms in Norway.

## Materials and methods

### Experimental design and diets

**Fig. 1.**
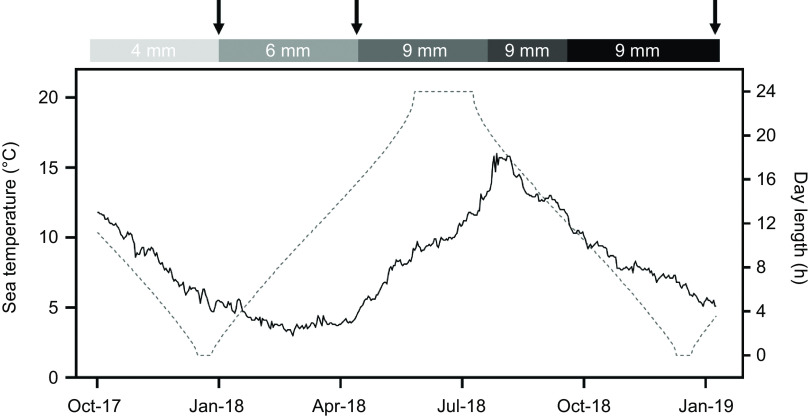
Daily sea temperature (°C), day length (h of daylight) and pellet size (mm) during the feeding trial (October 2017 to January 2019). Black arrows indicate weighing points (sampling 1, sampling 2 and final sampling from left to right) and define the three feeding periods according to pellet size (period 1, October 2017 to January 2018, 4-mm pellet; period 2, January 2018 to April 2018, 6-mm pellet and period 3, April 2018 to January 2019, 9-mm pellet).

Atlantic salmon post-smolts with an average weight of 115 g were purchased from Marine Harvest Norway AS, acclimated to the environmental conditions at the Gildeskål Research Station (GIFAS) and fed a standard commercial diet for this size (Biomar AS) prior to initiating the feeding trial. Fish with a mean initial weight of approximately 275 g were then randomly distributed into twelve outdoor sea cages (125 m^3^:5 × 5 × 5 m), with 190 fish per cage. The feeding trial was conducted from October 2017 to January 2019 (Fig. 1). Water salinity (measured at a depth of 3 m), O_2_ and temperature (average of measurements taken at 1, 2 and 3 m depth) were recorded daily. Temperatures ranged from 3°C (winter) to 16°C (summer), with an average of 7°C across the whole period. A naturally occurring cardiomyopathy syndrome (CMS) outbreak was diagnosed towards the end of the trial (September 2018). Mortality was recorded throughout the experiment.

Dietary treatments consisted of four experimental diets fed to triplicate groups of fish and formulated to test increasing dietary levels of EPA and DHA (approximately 1:1 ratio; 10, 13, 16 and 35 g/kg feed corresponding to 1·0, 1·3, 1·6 and 3·5 % of the diet). Fish were fed *ad libitum* once (autumn and winter periods, as well as during the CMS outbreak) or twice (spring and summer) per day. Three different pellet sizes (4, 6 and 9 mm) and specific formulations of experimental feeds (five separate feed productions) were used to meet the size and dietary requirements of the fish at the different stages (Fig. 1). Diets were formulated to be isoproteic, isolipidic and isoenergetic within each production and specifically designed to achieve a minimum amount of 50 mg of astaxanthin per kg of feed. The types of FM and FO (South American/North Atlantic) in the diets were adjusted according to the availability at the time of feed production. All diets were formulated to include approximately the same amount of FM within each feed production, except for the first period where the 3·5 % diet contained higher amounts compared with the other diets. EPA and DHA levels for the different dietary treatments slightly differed within each feed production. A summary of selected formulation (FO and FM) and chemical composition (energy, protein and fat) as well as EPA + DHA levels (g/100 g and % of total FA) in the diet for each feed production is shown in Table 1. A detailed diet formulation and chemical composition of the diets used in the last period of the trial is shown in Table 2 and all diets in online Supplementary Tables S1–S4. The FA composition of the diets corresponding to the last period of the trial is presented in Table 3. The designation of the diets for the different dietary treatments (1·0, 1·3, 1·6 and 3·5 %) refers to the weighted average amounts of EPA and DHA in terms of feed given for the whole trial.

**Table 1.** Summary of dietary fish oil (FO) and fish meal (FM) levels in feed, analysed chemical composition (protein and fat) and measured EPA + DHA levels (g/100 g feed and percentage of total fatty acids, TFA) of all the five experimental feeds produced during the trial

	Dietary EPA + DHA levels (%)			
	1·0	1·3	1·6	3·5
October 2017 to January 2018†				
FO*	2·3	4·8	6·3	9·5
FM*	15·0	15·0	15·0	41·5
Protein, crude	42·9	43·2	43·0	45·2
Fat, crude	27·9	28·3	28·3	27·7
EPA + DHA†	0·9	1·2	1·5	2·9
EPA + DHA in % of TFA	3·4	4·5	5·7	11·1
January 2018 to April 2018‡				
FO*	3·4	5·4	6·8	11·8
FM*	11	10	10	10
Protein, crude	39·5	39·5	39·4	39·5
Fat, crude	30·4	30·4	30·4	30·4
EPA + DHA†	1·1	1·1	1·3	2·4
EPA + DHA in % of TFA	4·3	4·3	4·6	8·5
April 2018 to August 2018§				
FO*	4·3	5·8	7·3	15
FM*	5	5	5	5
Protein, crude	36·2	36·2	36·2	36·2
Fat, crude	34·7	34·7	34·7	34·6
EPA + DHA*	1·1	1·4	1·7	3·3
EPA + DHA in % of TFA	3·3	4·3	5·2	10·3
August 2018 to September 2018‖				
FO*	3·8	5·1	6·4	14·2
FM*	5	5	5	5
Protein, crude	33·5	33·6	33·6	33·5
Fat, crude	38·2	38·2	38·2	37·7
EPA + DHA*	1·0	1·4	1·7	3·4
EPA + DHA in % of TFA	2·9	3·9	4·8	9·6
September 2018 to January 2019¶				
FO*	4·4	6·1	7·9	18·2
FM*	5	5	5	5
Protein, crude	33·4	33·4	33·4	33·5
Fat, crude	38·7	38·7	38·6	38·4
EPA + DHA*	1·0	1·4	1·7	3·7
EPA + DHA in % of TFA	2·8	3·8	4·8	10·4

* g/100 g feed.

† October 2017 to January 2018, 4-mm pellet, fish weight approximately 200 g.

‡ January 2018 to April 2018, 6-mm pellet, fish weight approximately 500 g.

§ April 2018 to August 2018, 9-mm pellet, fish weight approximately 1000 g.

‖ August 2018 to September 2018, 9-mm pellet, fish weight approximately 2500 g.

¶ September 2018 to January 2019, 9-mm pellet, approximately 3000 g.

**Table 2.** Detailed formulation and chemical composition of the experimental diets used during the last part of the trial (9-mm pellet; September 2018 to January 2019)

Formulation*	Dietary EPA + DHA levels (%)			
	1·0	1·3	1·6	3·5
Rapeseed oil, crude†	31·0	29·3	27·5	16·9
Guar meal‡	14	14	14	14
Soya protein concentrate	11·8	12·0	12·2	12·6
Wheat†	10·9	10·9	10·9	10·9
Fish oil§	4·4	6·1	7·9	18·2
Wheat gluten‖	7·8	7·7	7·6	6·5
Fish meal§	5	5	5	5
Maize gluten¶	5	5	5	5
Pea protein**	5	5	5	6
Monocalcium phosphate	1·3	1·3	1·3	1·3
Vitamin and mineral premix††	1·6	1·6	1·6	1·6
Amino acids‡‡	0·99	0·99	0·98	0·90
Lucatin pink CWD, 20 %	0·03	0·03	0·03	0·03
Water change	1·07	1·07	1·06	1·03
Chemical composition (analysed)				
Moisture	6	6	6	6
Energy, crude (MJ/kg)	26·2	26·2	26·2	26·1
Protein, crude	33·4	33·4	33·4	33·5
Fat, crude	38·7	38·7	38·6	38·4
Ash	4·4	4·4	4·4	4·3

* g/100 g feed.

† Denmark.

‡ India.

§ Peru/Denmark.

‖ EU.

¶ Ukraine.

** China.

†† Sweden.

‡‡ Germany/Korea/China.

**Table 3.** Fatty acid composition (% of total fatty acids) of the experimental diets used during the last part of the trial (9-mm pellet; September 2018 to January 2019)

	Dietary EPA + DHA levels (%)			
	1·0	1·3	1·6	3·5
14:0	0·9	1·1	1·4	2·8
16:0	7·0	7·2	7·5	9·6
18:0	2·8	2·7	2·7	3·0
20:0	0·7	0·6	0·6	0·5
Σ SFA*	12·6	13·1	13·3	17·0
16:1 *n*-7	1·0	1·3	1·8	3·6
18:1 *n*-7	2·8	3·0	3·0	3·2
18:1 *n*-9	46·5	45·8	43·8	33·0
20:1 *n*-9	1·6	1·9	2·4	4·2
20:1 *n*-11	0·3	0·4	0·5	1·1
22:1 *n*-9	0·2	0·2	0·2	0·3
22:1 *n*-11	0·6	0·9	1·3	3·1
24:1 *n*-9	0·2	0·2	0·2	0·2
Σ MUFA†	53·2	53·7	53·1	48·8
16:2 *n*-6	0·1	0·1	0·2	0·3
18:2 *n*-6	20·0	18·7	16·9	12·5
18:3 *n*-3	8·1	8·0	7·7	5·1
20:4 *n*-3	0·1	0·2	0·3	0·7
20:5 *n*-3	1·5	2·0	2·4	5·1
22:5 *n*-3	0·2	0·3	0·3	0·7
22:6 *n*-3	1·4	1·8	2·3	5·0
EPA + DHA	2·8	3·8	4·7	10·2
Σ PUFA	31·6	31·4	30·4	30·1
Σ *n*-3‡	11·5	12·5	13·3	17·2
Σ *n*-6§	20·2	19·0	17·2	13·1
*n*-6/*n*-3	1·8	1·5	1·3	0·8

* Includes 15:0. 17:0. 22:0. 24:0.

† Includes 16:1 *n*-5, 16:1 *n*-9, 17:1 *n*-7, 18:1 *n*-11, 20:1 *n*-7, and 22:1 *n*–7.

‡ Includes 20:3 *n*-3.

§ Includes 18:3 *n*-6.

### Sampling

At the beginning of the trial, fish were individually weighed and randomly distributed into the experimental sea cages (approximately 190 per cage, three replicates per treatment). Whole-body samples of fish (three independent replicas of pools of five fish randomly selected from the stock) were taken at the start of the trial and at each shift in pellet size of the feed; after 3 months (January 2018, end of the 4-mm pellet period), 6 months (end of the 6-mm pellet period) and at the final sampling (pools of five fish per cage) and stored at −40°C for chemical and FA composition, as well as whole-body nutrient retention analyses. The fish from all cages were bulk weighed at each change of pellet size. At the end of the trial (January 2019), fifteen fish per cage were randomly killed by anaesthetic overdose (Tricaine Pharmaq, 0·3 g/l) and blood samples were taken from the caudal vein. The blood was centrifuged for 7 min at 2500 ***g*** to separate the plasma fraction, which was then stored at −80°C. These fifteen fish were also used for biometric, fillet quality and welfare scoring measurements, as well as X-ray analyses. A further fifteen fish per cage were killed for dissecting samples from muscle (Norwegian Quality Cut) and skin. From those, five fish per cage were used for dissecting liver, middle intestine and distal intestine, frozen at −80°C and stored for later analysis of lipid composition. Five more samples per cage of liver, skin (left side on the area posterior of the dorsal fin and above the lateral line), middle intestine, distal intestine and heart (including atrium and ventricle) were fixed in 10 % formalin and stored at 4°C prior to processing. Five samples per cage of hearts were excised and cut into suitable pieces for storage in RNAlater stabilisation reagent (Qiagen Nordic) for RNA analysis. Additionally, feed samples were frozen at −40°C for chemical and FA composition analyses. All animal handling procedures complied with the National Guidelines for Animal Care and Welfare published by the Norwegian Ministry of Education and Research (Norwegian Food Safety Authority (FOTS), approval 16059).

### Growth, feed intake parameters and fatty acid retention calculations

Fish weights were recorded as previously stated and growth rates were calculated as follows, based on average values per cage:

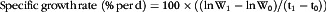




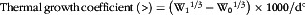

where W_0_ is the start weight (g) and W_1_ is the final weight (g) at times t_1_ and t_0_, respectively, and d° is the sum of day degrees.

The feed conversion ratio (FCR) was calculated based on the recorded feed intake and biomass increase per cage as follows:

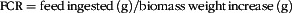




The specific feeding rate (SFR) and condition factor were calculated using the formulas:

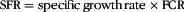




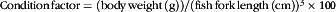




The apparent FA retention was calculated per cage and for three feeding periods (period 1 (P1), October 2017 to January 2018, 4-mm pellet; period 2 (P2), January 2018 to April 2018, 6-mm pellet and period 3 (P3), April 2018 to January 2019, 9-mm pellet) according to the following general formula:

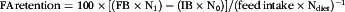

where FB and IB represent the final (F) and initial (I) biomass, and N represents the concentration of FA in fish (where 1 is the final and 0 the initial sampling day) or diet. The final biomass was corrected for mortality for all experimental periods.

### Fat content, fatty acid composition and chemical composition analyses

Total lipids and FA composition were analysed in the diets, whole body, muscle (Norwegian Quality Cut), liver, skin, middle intestine and distal intestine as previously described by Folch *et al.* ^(20)^ and Mason and Waller^(21)^, respectively. Lipids were extracted from the five productions of experimental diets and homogenised tissues (whole body, fillet, liver, skin, middle intestine and distal intestine). The chloroform–methanol phase obtained from all samples after the Folch extraction was dried under N_2_ gas, and residual lipid was trans-methylated overnight with 2′-2′-dimethoxypropane, methanolic HCl and benzene at room temperature. Methyl esters were separated and analysed using a gas chromatograph (Hewlett Packard 6890; HP) equipped with a split injector using an SGE BPX70 capillary column (length, 60 m; internal diameter, 0·25 mm and film thickness, 0·25 μm; SGE Analytical Science) and a flame ionisation detector. Results were further analysed using HP ChemStation software. Helium was used as the carrier gas, and the injector and detector temperatures were both set at 280°C. The oven temperature was increased from 50 to 180°C at a rate of 10°C/min and then increased to 240°C at a rate of 0·7°C/min. Individual FA methyl esters were identified by referring to previously characterised standards. The relative amounts of each FA were expressed as a percentage of the total amount of FA in the analysed sample, and the absolute amount of FA per gram of tissue was calculated using C23:0 methyl ester as the internal standard.

The chemical composition of the diets and whole-body fish samples was analysed as follows. DM was determined by gravimetric analysis following drying at 105°C for 16 h. Ash content was measured based on mass change after combustion in a muffle furnace at 550°C for 16 h. Measurement of total N_2_ content was performed using an elemental analyser (Flash 2000; Thermo Fisher Scientific), and the data were used to calculate the protein content of the sample based on *n* × 6·25. Gross energy was determined by isoperibolic bomb calorimetry in a Parr 6200 oxygen bomb calorimeter (Parr Instrument Company). Carbohydrate content was calculated using the difference with the previously mentioned calculated parameters (based upon nutrient percentages subtracted from 100 % of feed DM).

### Biochemical analysis of plasma parameters

Plasma samples were examined using diagnostic reagents for quantitative determination of glucose, creatine kinase (CK), aspartate aminotransferase and alanine aminotransferase using an ABX Pentra 400 hematology analyzer (Horiba ABX SAS). All analyses were conducted according to the manufacturer’s protocol. The measurement range was 4–1800 U/l for aspartate aminotransferase and alanine aminotransferase and 8–4500 U/l for CK.

### Histology preparation, X-ray and pathology evaluation

Embedding, sectioning and staining of the tissue samples were performed at the Norwegian Veterinary Institute. In brief, tissue samples were dehydrated in ethanol, equilibrated in xylene and embedded in paraffin according to standard histological techniques. Sections of approximately 5 µm were cut and stained with haematoxylin and eosin for liver, heart, middle intestine and distal intestine, and with Alcian blue/periodic acid–Schiff for skin. The sections were subsequently scanned (Aperio ScanScope AT Turbo slice scanner), and the resulting digital images from liver, middle intestine and distal intestine were subjected to a blind histopathological evaluation on screen (Aperio Image Scope v12.4; FastStone Image Viewer 7.0). A total of sixty samples per tissue were examined (five samples per cage). Tissue samples were evaluated for pathology and other systematic variations in tissue morphological features. Liver samples were examined to determine the degree of microvesicular and/or macrovesicular steatosis using a semi-quantitative scoring scale from 1 to 5, where 1 represents sparse vacuolation and 5 represents severe fatty change. Samples from the middle and distal intestine underwent a simple histological evaluation to describe general morphological features, such as folding of intestine mucosa and enterocyte vacuolisation. Skin images were evaluated using an artificial intelligence model (Aiforia; Aiforia Technologies Oy) designed to identify changes in mucous cells, dermis, epidermis, connective tissue, pigmentation and scales as previously described^(22)^. A total of fifty samples were included in the analysis (three to five samples per cage). Slides from heart (atrium/ventricle) were examined by light microscopy, and histopathological changes were scored by an experienced histopathologist from the Faculty of Veterinary Medicine (Norwegian University of Life Sciences) using previously described criteria^(23)^. A total of sixty samples were included in the analysis (five fish per cage/fifteen fish per diet group). For the X-ray analysis, fish were filleted, and the vertebral bones were frozen for radiographic analysis at the Nofima X-ray Laboratory in Sunndalsøra, Norway. Samples were subsequently X-rayed using a semi-digital system with reusable image plates, with two to three spines per image. The resulting images were examined to identify and classify specific pathologies. A total of twenty-two to twenty-five samples per diet were examined.

### Evaluation of welfare indicators and fillet quality

Fish were evaluated for external (eye cataract, skin lesions, snout damage and fin damage, including dorsal, pectoral and caudal fins) and internal (melanisation in visceral adipose tissue and heart, heart shape, stomach and intestinal inflammation and fat accumulation in the liver) welfare indicators using a scoring system developed by Nofima^(24)^ and Biomar (online Supplementary Table S5). Each of the welfare indicators was given a score between 0 and 3 (external) or 1 and 2, 3 or 4 (internal), where the lowest value represents good and the highest represents bad condition. External and internal evaluations were performed by the same person. Additionally, scoring values from external or internal indicators were transformed to equivalent ordinal categories and mapped together on a radar plot, and the resulting area was calculated and used as a summarised index (external welfare index; internal welfare index) according to the general formula:

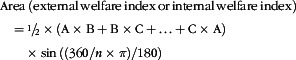

where the capital letters represent each of the welfare indicators and ‘*n*’ represents the number of welfare indicators included in the external or internal evaluation.

Fillet quality analyses included fillet colour from anterior and posterior parts of the fillet, number of melanin spots, percentage liquid loss after freezing and thawing and astaxanthin and idoxanthin levels. Colour was visually evaluated under standardised light conditions (Salmon Colour Box, Skretting) using DSM SalmonFan above the lateral line anterior to the dorsal fin and between the caudal part of the dorsal fin and the gut (Norwegian Quality Cut). All measurements were taken by the same skilled person (blind analyses with fillets provided in random order). Melanin discolouration in the cranioventral fillet was visually assessed in terms of the number of dark spots as previously described^(25)^. Liquid loss was determined gravimetrically by freezing 50 g cutlets below the posterior part of the dorsal fin at −20°C for 1 month and weighing the cutlets after thawing at 2°C. Astaxanthin and idoxanthin levels were analysed in pools of Norwegian Quality Cut cutlets according to the method described by Bjerkeng *et al*.^(26)^ In brief, samples were homogenised using a 1:1:3 mixture of distilled water, methanol and chloroform. Then, after centrifugation (10 min at 3000 rpm), 5 ml of the chloroform phase was removed and re-dissolved in acetone/*n*-hexane/methanol (20:80:0·1), filtered through a 0·45-μm filter and analysed isocratically by a HPLC (Spherisorb S5-CN nitrile column; PhaseSep). The extraction procedure was repeated twice. Standard samples were prepared from crystalline all-*E*-astaxanthin (Hoffmann-La Roche Ltd.), and their concentrations were measured spectrophotometrically (UV-260) using a molar absorptivity E1 %, 1 cm = 2100 (λmax = 470 nm) in *n*-hexane containing 4·5 % chloroform. The percentages of astaxanthin and idoxanthin were calculated from chromatogram areas and corrected for differences in extinction coefficients (E1 %, 1 cm)^(27,28)^.

### Statistical analyses

Differences among dietary treatments were assessed using GraphPad Prism 8 (www.graphpad.com) and JMP software version 11.2.1 (SAS Institute Inc.). Data normality and homoscedasticity were assessed using Shapiro–Wilk and Levene’s tests, respectively. Statistical significance was evaluated by one-way ANOVA followed by Tukey’s honest significant difference *post hoc* test. Heart histoscores (categorised as 0–4) were analysed by ordinal logistic regression using Stata 15 using the 1 % EPA and DHA group as the baseline. Differences with *P*-values < 0·05 were considered statistically significant. Cages were considered as experimental units (*n* 3) for all analyses except for fillet quality, plasma analyses and heart lesions where values from individual fish (*n* 45) were used. Additionally, relative FA composition data of salmon tissues were analysed using Unscrambler X version 10.3 (CAMO). Multivariate principal component analysis was performed for each data matrix of the relative FA compositions. Score plots from principal component analysis were used to explore the main trends and groupings in the data, and their respective correlation loadings reveal variables contributing to sample groupings. Linear regression models were used to evaluate the relationship between FA tissue content and EPA and DHA levels in the feed.

## Results

### Fish performance, tissue lipid content and feed intake

Fish weight, length, condition factor, specific growth rate, TGC, feed intake, FCR, SFR and total lipid content in different tissues are presented in Table 4. Final weight, length, specific growth rate and TGC were significantly higher in the fish fed the diet containing 3·5 % EPA and DHA compared with those fed the other diets. The 3·5 % EPA and DHA group showed approximately 12, 8 and 8 % higher final weights as well as 6, 4 and 4 % higher growth (TGC) at the end of the trial compared with the 1·0, 1·3 and 1·6 % groups, respectively. However, no significant differences were observed for growth rates, weight or condition factor (data not shown) at earlier sampling times when fish were approximately 600 and 1000 g, highlighting the relevance of long-term trials to reveal all the potential effects of dietary *n*-3 PUFA on growth performance. Moreover, the total lipid content in the liver was significantly affected by dietary treatments and was approximately 45 % lower in the fish fed the 3·5 % EPA and DHA diet compared with all other dietary groups, whereas no differences were found in muscle, skin, middle intestine or distal intestine. Even though no significant differences were recorded, the lipid content in distal intestine in 3·5 % group appeared to be approximately 30 % higher compared with the 1 % group.

**Table 4.** Growth performance, biometric data and total lipid content (g lipid/100 g tissue) in tissues of salmon fed the four experimental diets (1, 1·3, 1·6 or 3·5 % of EPA and DHA) (Mean values with their standard errors)

	Dietary EPA + DHA levels (%)								*P* (ANOVA)
	1·0		1·3		1·6		3·5		
	Mean	sem	Mean	sem	Mean	sem	Mean	sem	
Initial weight (g)	275·4	1·37	275·2	2·88	276·4	0·63	277·1	0·66	0·835
S1 weight (g)	590·5	9·69	578·9	11·68	594·7	9·11	604·3	20·14	0·624
S2 weight (g)	982·3	16·08	981·6	21·77	969·4	5·95	1011·1	18·97	0·399
Final weight (g)	4748·5^a^	33·40	4938·0^a^	85·55	4963·6^a^	68·43	5364·6^b^	56·93	<0·001
Final length (cm)	70·4^a^	0·052	70·8^a^	0·395	69·7^a^	0·495	72·8^b^	0·556	0·004
CF	1·5	0·020	1·5	0·020	1·5	0·007	1·5	0·015	0·357
SGR	0·68^a^	0·003	0·69^a^	0·005	0·69^a^	0·003	0·70^b^	0·002	0·022
TGC	3·1^a^	0·016	3·1^a^	0·031	3·1^a^	0·023	3·3^b^	0·017	0·001
Feed intake (kg/d)	1·9	0·05	1·9	0·05	1·8	0·10	2·0	0·07	0·328
FCR	1·11	0·007	1·10	0·006	1·10	0·014	1·11	0·008	0·765
SFR	0·75^a^	0·007	0·75^a^	0·005	0·75^a^	0·01	0·78^b^	0·005	0·039
Lipid content									
Liver	10·4^a,b^	0·80	12·4^a^	1·8	10·2^a,b^	0·57	6·0^b^	0·45	0·016
Muscle	20·4	0·41	20·9	1·25	20·4	0·72	20·0	0·26	0·859
Skin	17·3	1·82	14·4	1·10	17·3	1·78	18·1	1·29	0·391
Middle intestine	5·9	0·95	5·0	0·25	5·1	0·38	4·9	0·74	0·681
Distal intestine	3·9	0·54	2·7	0·22	3·8	0·20	5·7	1·25	0·088

S1, sampling 1; S2, sampling 2; CF, condition factor; SGR, specific growth rate; TGC, thermal growth coefficient; FCR, feed conversion rate and SFR, specific feeding rate.

Data are shown as mean ± sem (*n* 3 cages).

Different letters within each row indicate significant differences between values determined using a one-way ANOVA followed by Tukey’s *post hoc* test.

**Fig. 2.**
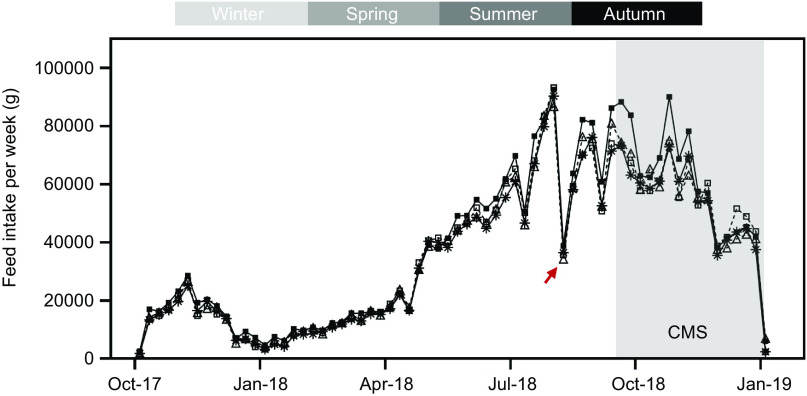
Weekly feed intake (g) throughout the whole feeding trial. Mechanical delousing and a cardiomyopathy syndrome (CMS) outbreak are indicated by a red arrow and grey background, respectively. Statistical differences are not shown in the graph. 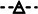, 1 %; 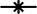, 1·3 %; 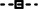, 1·6 %; 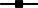, 3·5 %.

Feed intake was higher in the fish fed the 3·5 % EPA and DHA diet, although there were no significant differences among the feeding groups (*P* = 0·276). Furthermore, an FCR of 1·1 and SFR of approximately 0·7 were observed for all experimental groups. In particular, the SFR in the 3·5 % EPA and DHA group was significantly higher compared with the others (*P* = 0·039). Throughout the trial, feed intake showed a similar trend for all the groups and followed a typical seasonal pattern for the geographic region, with increased values at higher water temperatures and prolonged daylight during spring and summer, and lower values at lower temperatures and reduced daylight during autumn and winter (Fig. 2). Interestingly, the 3·5 % EPA and DHA group showed a better recovery of feed intake compared with the other groups after treatment for sea lice (indicated by a red arrow) as well as after the onset of the CMS outbreak (September 2018). However, no significant differences were observed at any of the different timepoints evaluated.

### Whole-body total lipid content, fatty acid composition and apparent retention

Whole-body total lipid content and FA profiles from all experimental groups are presented in Table 5. The whole-body total fat content was approximately 27 % (gram fat per 100 g body weight) of total body weight and showed no significant differences between dietary treatments reflecting the same levels of fat in the different diets. Moreover, the total FA composition showed increased levels of SFA and PUFA, concomitant with the increase in EPA and DHA in the diets and was significantly higher in the 3·5 % EPA and DHA group compared with all the other groups (*P* < 0·0001). Among PUFA, *n*-3 PUFA, specifically EPA and DHA, were significantly higher in the fish fed the 3·5 % EPA and DHA diet. Particularly, the percentage EPA and DHA of total FA was more than double in these fish compared with those fed the diets containing 1·0 % and 1·3 % EPA and DHA (9·6 % *v*. 3·5 % and 4·1 %, respectively) and was almost double that of the fish fed 1·6 % EPA and DHA diet (9·6 % *v*. 5 %). The opposite result was found for MUFA, total *n*-6 PUFA and the *n*-6/*n*-3 PUFA ratio, and levels decreased concomitantly with the increase in EPA and DHA in the diet. Fish fed the 3·5 % EPA and DHA diet exhibited the lowest percentages of these FA compared with all the other dietary treatments (*P* < 0·0001).

**Table 5.** Total lipid content (g lipid/100 g tissue) and fatty acid composition (% of total FA) in the whole body of Atlantic salmon fed the four experimental diets (1, 1·3, 1·6 or 3·5 % EPA and DHA in the diet) (Mean values with their standard errors)

	Dietary EPA + DHA levels (%)								*P* (ANOVA)
	1		1·3		1·6		3·5		
	Mean	sem	Mean	sem	Mean	sem	Mean	sem	
Lipid content	26·5	0·4	27·0	0·77	27·0	0·43	27·0	0·30	0·8418
14:0	0·9^a^	0·00	1·1^b^	0·01	1·3^c^	0·00	2·5^d^	0·01	<0·0001
16:0	7·8^a^	0·08	8·1^a^	0·15	8·6^b^	0·04	10·6^c^	0·05	<0·0001
18:0	2·4^a^	0·03	2·4^a^	0·03	2·4^a^	0·07	2·8^b^	0·03	0·0005
Σ SFA*	12·2^a^	0·13	12·6^a^	0·18	13·4^b^	10·14	16·9^c^	0·06	<0·0001
18:1 *n*-9	47·4^a^	0·08	46·1^b^	0·11	44·7^c^	0·06	36·0^d^	0·10	<0·0001
18:1 *n*-7	2·7	0·09	2·7	0·08	2·7	0·04	2·9	0·06	0·0966
20:1 *n*-11	0·6	0·03	0·6	0·02	0·6	0·03	0·6	0·01	0·2275
20:1 *n*-9	2·4	0·02	2·4	0·04	2·4	0·02	2·3	0·05	0·3928
22:1 *n*-7	0·6^a^	0·02	0·7^a^	0·01	0·7^a^	0·02	0·8^b^	0·01	0·0007
22:1 *n*-11	0·3^a^	0·01	0·4^a^	0·00	0·4^a^	0·01	0·9^b^	0·05	<0·0001
Σ MUFA†	56·3^a^	0·10	55·2^b^	0·08	54·2^c^	0·11	47·8^d^	0·09	<0·0001
18:2 *n*-6	17·3^a^	0·07	17·0^a^	0·10	16·4^b^	0·03	13·8^c^	0·05	<0·0001
20:2 *n*-6	1·2^a^	0·01	1·2^a^	0·03	1·1^a^	0·03	1·0^b^	0·01	0·0011
20:3 *n*-6	0·5^a^	0·02	0·4^a,b^	0·01	0·4^b^	0·02	0·2^c^	0·01	<0·0001
20:4 *n*-6	0·2^a^	0·00	0·2^b^	0·01	0·3^c^	0·00	0·5^d^	0·00	<0·0001
18:3 *n*-3	6·7^a^	0·08	6·7^a^	0·05	6·5^b^	0·03	5·4^c^	0·02	<0·0001
20:3 *n*-3	0·4^a,b^	0·01	0·5^a,b^	0·01	0·5^a^	0·02	0·4^b^	0·00	0·0432
20:5 *n*-3	1·5^a^	0·01	1·8^b^	0·02	2·2^c^	0·02	4·4^d^	0·01	<0·0001
22:5 *n*-3	0·6^a^	0·01	0·7^b^	0·02	0·9^c^	0·01	1·9^d^	0·03	<0·0001
22:6 *n*-3	2·0^a^	0·05	2·3^b^	0·01	2·8^c^	0·01	5·1^d^	0·02	<0·0001
EPA + DHA	3·5^a^	0·05	4·1^b^	0·00	5·0^c^	0·03	9·6^d^	0·02	<0·0001
Σ PUFA‡	31·0^a^	0·17	31·6^b^	0·12	31·7^b^	0·06	33·8^c^	0·05	<0·0001
Σ *n*-3	11·5^a^	0·10	12·3^b^	0·08	13·2^c^	0·05	17·8^d^	0·03	<0·0001
Σ *n*-6	19·5^a^	0·06	19·2^b^	0·07	18·5^c^	0·02	15·9^d^	0·04	<0·0001
*n*-6/*n*-3	1 7^a^	0 01	1 6^b^	0 01	1 4^c^	0 0	0 9^d^	0 00	<0·0001

* Includes 15:0, 17:0, 20:0, 22:0, 24:0.

† Includes 14:1 *n*-5, 16:1 *n*-5, 16:1 *n*-9, 17:1 *n*-7, 18:1 *n*-11, 20:1 *n*-7, 22:1 *n*-9, 24:1 *n*-9.

‡ Includes 16:2 *n*-3, 16:2 *n*-6, 16:3 *n*-4, 18:3 *n*-6, 18:4 *n*-3, 20:4 *n*-3.

Data are shown as mean ± sem (*n* 3 cages).

Different letters within each row indicate significant differences between values determined using a one-way ANOVA followed by Tukey’s *post hoc* test.

Apparent FA retentions were calculated to determine the actual amount of the dietary EPA and DHA consumed that was deposited in the whole body for all groups. The deposition of FA in salmon may vary depending on the feed composition and developmental stage of the fish; thus, FA retention values were calculated for three different time periods according to pellet size and differences in feed formulation (P1, P2 and P3). The values for 20:5 *n*-3, 22:5 *n*-3 (DPA) and 22:6 *n*-3 in the three different periods are presented in Table 6. The retentions of 20:5 *n*-3, 22:5 *n*-3 and 22:6 *n*-3 (*P* = 0·06) during P1 were affected by the dietary levels of EPA and DHA, with the 3·5 % EPA and DHA group exhibiting higher values compared with all the other dietary treatments. Conversely, this trend was reversed during P2 and showed a tendency towards lower 20:5 *n*-3 retention with increased dietary inclusion of EPA and DHA, and significantly lower retention for 22:6 *n*-3 in the 3·5 % EPA and DHA group. In particular, the retention values for the 22:6 *n*-3 were less than half in the fish fed the 3·5 % EPA and DHA diet compared with those fed 1·0 % and 1·3 % EPA and DHA. No significant changes were observed for 22:5 *n*-3 when comparing any of the different dietary groups in this period. During P3, a lower 22:6 *n*-3 retention tendency was not evident and was only significant compared with fish fed the 1·6 % EPA and DHA diet.

**Table 6.** Apparent retention (% deposited in the whole body relative to feed intake) of EPA (20:5 *n*-3), DPA (22:5 *n*-3) and DHA (22:6 *n*-3) during three different periods (P1, P2 and P3) according to pellet size and differences in feed formulation of Atlantic salmon fed the four experimental diets (1, 1·3, 1·6 or 3·5 % EPA and DHA) (Mean values with their standard errors)

		Dietary EPA + DHA levels (%)								*P* (ANOVA)
		1·0		1·3		1·6		3·5		
		Mean	sem	Mean	sem	Mean	sem	Mean	sem	
P1	20:5 *n*-3	21·33^a^	1·93	20·17^a^	2·82	25·63^a^	1·84	32·83^b^	1·46	0·01
	22:5 *n*-3	106·33^a^	19·85	82·33^a^	3·22	87·87^a^	4·54	154·47^b^	11·17	0·006
	22:6 *n*-3	35·20	6·57	36·00	7·35	36·87	5·19	58·83	3·49	0·06
P2	20:5 *n*-3	40·80	1·37	49·13	7·36	33·03	4·22	32·43	2·53	0·17
	22:5 *n*-3	107·27	6·61	119·50	14·98	65·23	17·90	113·10	8·92	0·09
	22:6 *n*-3	76·47^a^	3·32	69·57^a^	16·67	47·37^a,b^	13·88	26·20^b^	5·24	0·049
P3	20:5 *n*-3	54·33	1·62	54·10	2·36	59·40	2·79	53·10	0·57	0·26
	22:5 *n*-3	168·90	6·39	164·57	5·93	183·77	10·51	172·60	3·04	0·16
	22:6 *n*-3	72·80^a,b^	2·62	72·10^a,b^	2·01	84·30^a^	6·68	66·90^b^	0·45	0·049

Data are shown as mean ± sem (*n* 3 cages).

Different letters within each row indicate significant differences between values determined using a one-way ANOVA followed by Tukey’s *post hoc* test.

The different periods were as follows: P1, October 2017 to January 2018, 4-mm pellet; P2, January 2018 to April 2018, 6-mm pellet; and P3, April 2018 to January 2019, 9-mm pellet.

### Multivariate comparison and linear regression analysis of the fatty acid composition in different tissues

**Fig. 3.**
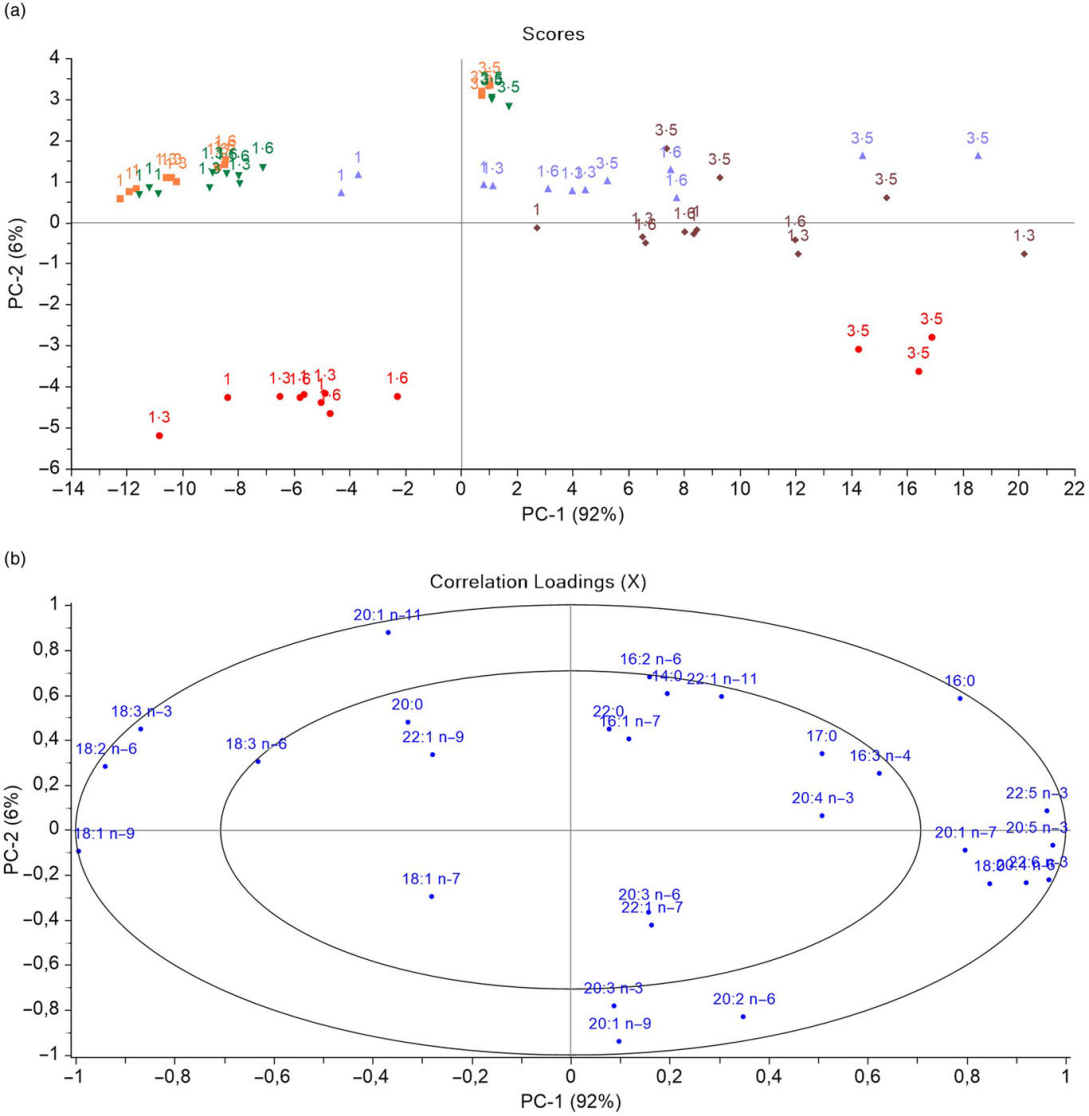
Scores (a) and correlation loadings (b) showing the relationships between the different tissues and the total fatty acid composition (% of total fatty acid) from Atlantic salmon fed the four experimental diets (1, 1·3, 1·6 or 3·5 % EPA and DHA in the diet). The plots show first principal component (PC1, 92 %) *v*. second principal component (PC2, 6 %), summarising 98 % of the variation. The scores in the plot indicate the percentage of dietary EPA and DHA (1, 1·3, 1·6 and 3·5 %) represented in different colours according to the type of sample analysed (muscle, liver, middle intestine, distal intestine and skin). 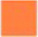, muscle; 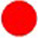, liver; 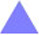, midgut; 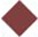, hindgut; 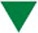, skin.

Multivariate principal component analysis and linear regression analyses, including FA composition data for muscle, liver, middle intestine, distal intestine and skin, were conducted to further evaluate the specific effects of increasing the dietary EPA and DHA levels (Fig. 3). The FA composition of all tissues (liver, skin, muscle, middle intestine and distal intestine) is shown in online Supplementary Tables S6–S10. Principal component analysis plot analysis revealed a clustering of samples with similar relative total FA compositions in the same area (Fig. 3(a)). The scores of samples belonging to the same tissue, particularly muscle, liver and skin, were located together and influenced by the dietary groups. In this regard, 92 % of the variation was explained by the first principal component (*x*-axis), which distributed the samples in a percentage-dependent manner along the axis from 1·0 % in the left quadrant to 3·5 % in the right quadrant. Scores from middle and especially distal intestine samples followed a similar trend but were less clearly affected by dietary treatment and showed no evident separation between groups. Separation between dietary groups was characterised by high percentages of the typical feed FA, including 18:1 *n*-9, 18:2 *n*-6 and 18:3 *n*-3, located towards the left quadrant and corresponding to samples with lower dietary EPA and DHA content (1·0 and 1·3 %), whereas samples from the 3·5 % EPA and DHA group, and from liver, were richer in 20:5 *n*-3, 22:5 *n*-3 and 22:6 *n*-3 (Fig. 3(b)). The second principal component separated liver samples (lower quadrant) from muscle and skin, which were clustered very closely, and middle and distal intestines.

**Fig. 4.**
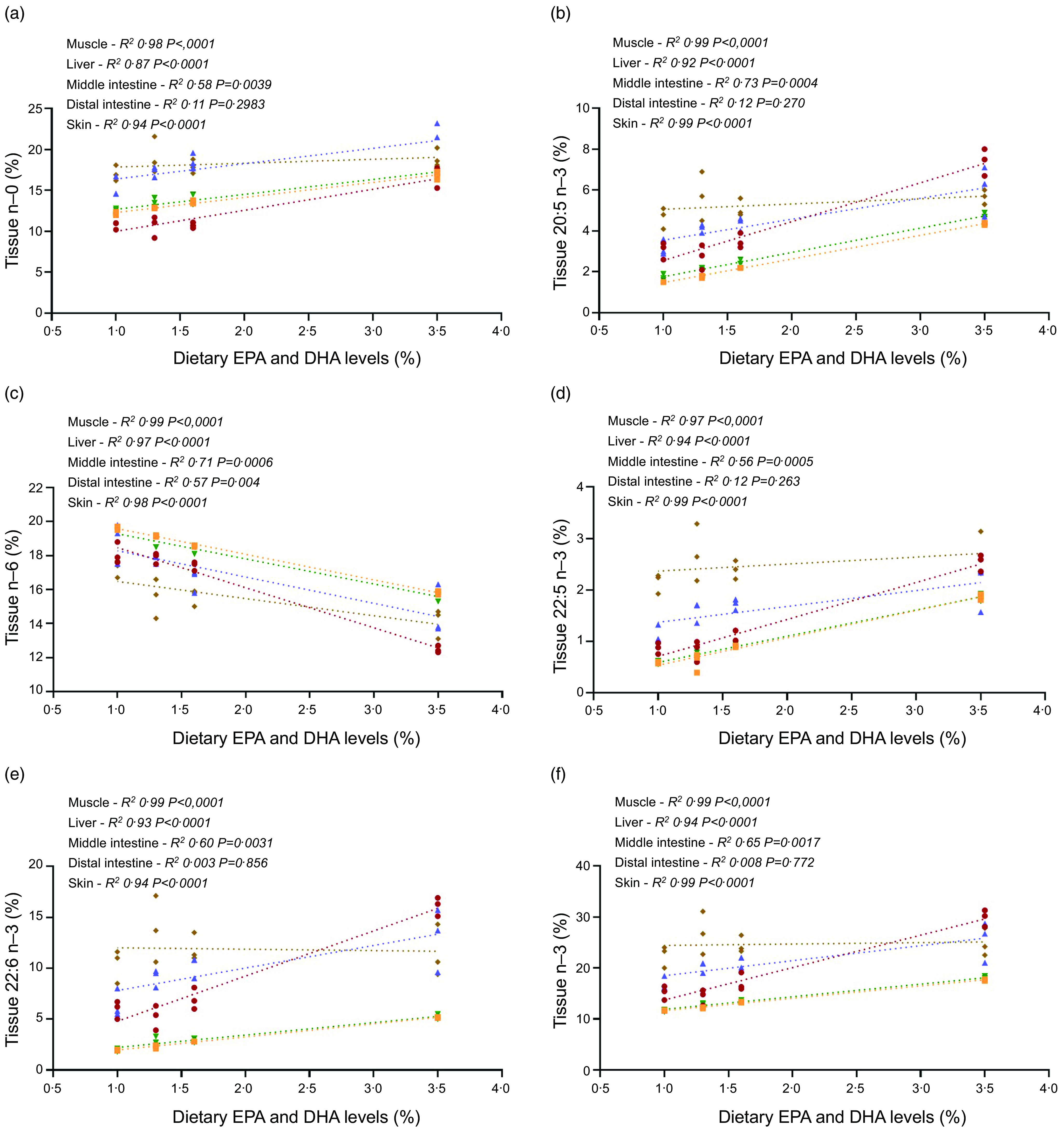
Regressions of dietary EPA and DHA and the respective content (% of identified fatty acids (FA) in the different tissues) of *n*-0 (a), *n*-6 (b), 22:6 *n*-3 (c), 20:5 *n*-3 (d), 22:5 *n*-3 (e) and *n*-3 PUFA (f) FA in muscle, liver, middle intestine, distal intestine and skin from Atlantic salmon fed the four experimental diets (1, 1·3, 1·6 or 3·5 % EPA and DHA).

Regression analyses showed that the percentages of SFA (*n*-0) in the muscle, liver, skin and middle intestine increased concomitantly with the increase in dietary FO. However, SFA levels were only moderately increased in the distal intestine (Fig. 4(a)). Similarly, muscle, skin, middle intestine and especially liver showed a gradual increase in *n*-3 PUFA levels with increasing dietary EPA and DHA levels (Fig. 4(f)), along with a corresponding decrease in *n*-6 PUFA levels (Fig. 4(b)). Among *n*-3 PUFA, muscle and skin showed almost identical values, with a linear increase in 20:5 *n*-3 (from 1·5 and 1·7 % to 4·3 and 4·7 %, respectively), 22:5 *n*-3 (from 0·6 to 1·9 % for both tissues) and 22:5 *n*-3 (from 2·0 to 5·2 % for both tissues) as the dietary EPA and DHA levels increased from 1 % to 3·5 %. Similar results were found in the middle intestine, with values ranging from 3·2 to 6·1 % for 20:5 *n*-3, 1·1 to 2·1 % for 22:5 *n*-3 and 6·4 to 13 % for 22:6 *n*-3. Moreover, liver samples exhibited approximately a 2·5-fold increase in 20:5 *n*-3, 22:5 *n*-3 and 22:6 *n*-3, reaching values of 7·4, 2·5 and 16·1 % of the total FA in the 3·5 % EPA and DHA dietary group. In the distal intestine, levels of all these FA were only moderately affected by the diets compared with the other tissues (Fig. 4(c)–(e)).

### Plasma parameters, mortality and impact of the cardiomyopathy syndrome natural outbreak

**Fig. 5.**
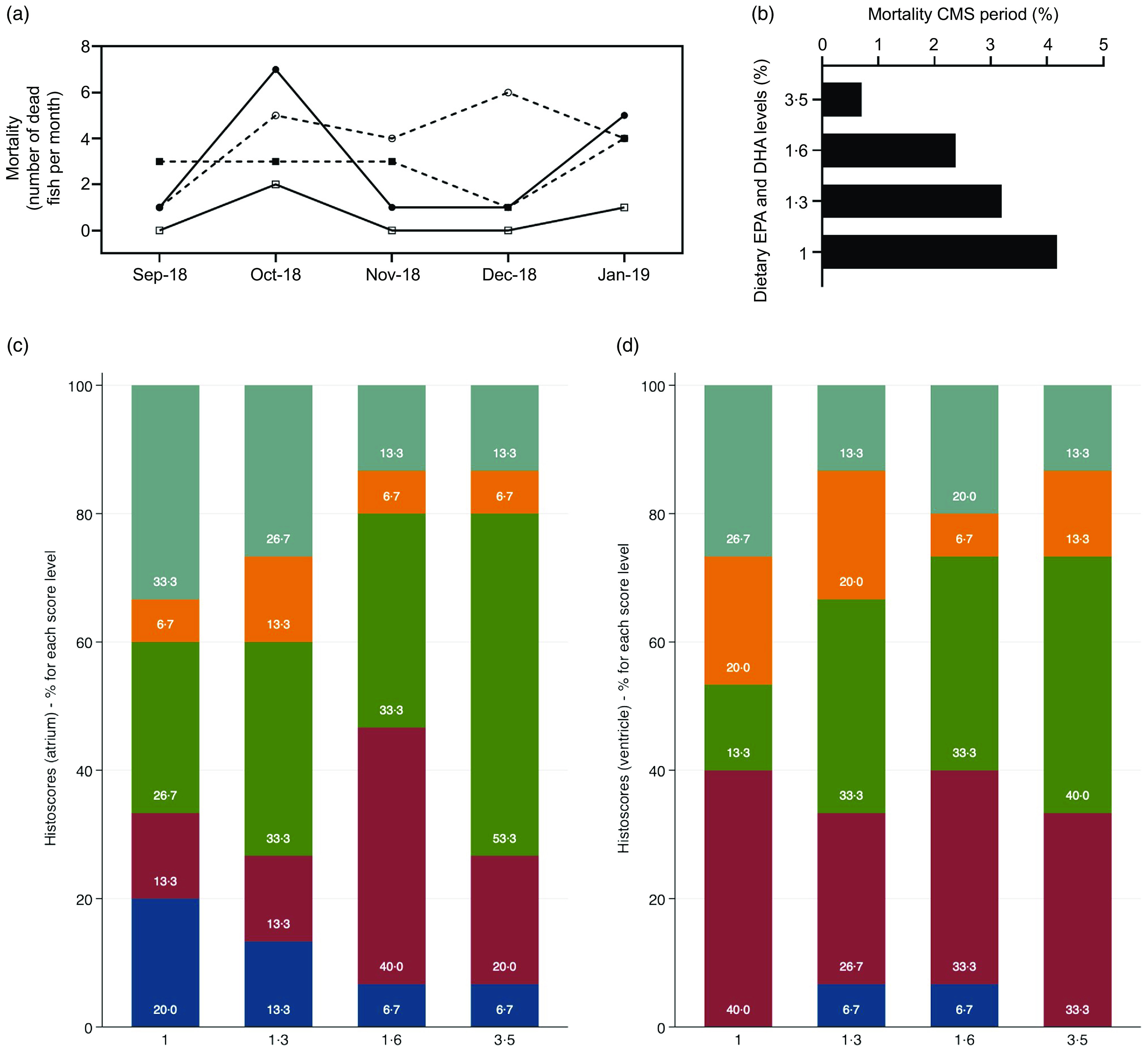
Monthly number of dead fish during the cardiomyopathy syndrome (CMS) outbreak (a), percentage of mortality for the whole CMS outbreak period (b) and heart histoscorings graded 0–4 (c, atrium; d, ventricle) of Atlantic salmon (a total number of sixty samples, five samples per cage) fed the four experimental diets (1, 1·3, 1·6 or 3·5 % of EPA and DHA). (a) 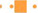, 1 %; 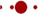, 1·3 %; 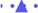, 1·6 %; 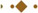, 3·5 %. (c and d) 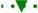, 0; 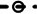, 1; 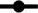, 2; 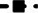, 3; 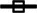, 4.

During the experiment, the average mortality of fish in all experimental groups was approximately 7·5 % and was not significantly influenced by diet. Predation (otters) was the main cause of death during the first half of the trial and most likely influenced the final mortality results. The number of dead fish was also analysed specifically for the CMS outbreak period in order to determine any potential difference in fish robustness among the dietary groups. Although the number of dead fish per cage during this period was not sufficient to perform a proper statistical analysis, there was a tendency towards lower number of dead fish due to this disease (confirmed by an experienced veterinarian) with increased dietary EPA and DHA levels, with the 3·5 % EPA and DHA group showing the lowest number of dead fish compared with the other dietary groups (Fig. 5(a) and (b)). Analysis of glucose, CK, aspartate aminotransferase and alanine aminotransferase in plasma revealed no significant differences in response to dietary treatments (Table 7). However, CK values showed a slight tendency to reduce concomitantly with the increase in dietary EPA and DHA. Nevertheless, there were no statistical differences among feeding groups for heart (ventricle and atrium) histoscores using the 1 % EPA and DHA group as a baseline (Fig. 5(c) and (d)), and RT-PCR analysis also showed no statistical differences in piscine myocarditis virus expression levels in the heart between the feeding groups (not shown).

**Table 7.** Selected biochemical plasma parameters of Atlantic salmon fed the four experimental diets (1, 1·3, 1·6 or 3·5 % EPA and DHA) (Mean values with their standard errors)

	Dietary EPA + DHA levels (%)								*P* (ANOVA)
	1·0		1·3		1·6		3·5		
	Mean	sem	Mean	sem	Mean	sem	Mean	sem	
Glucose (mmol/l)	5·3	0·2	4·9	0·33	5·1	0·1	5·4	0·2	0·445
CK (U/l)	15 392·1	3756·8	12 053·3	4697·0	10 812·8	1070·1	5881·3	606·0	0·253
ASAT (U/l)	347·7	21·1	359·0	48·7	364·1	33·2	326·7	47·5	0·908
ALAT (U/l)	6·3	0·7	6·5	0·3	6·1	1·3	7·3	1·7	0·883

CK, creatine kinase; ASAT; aspartate aminotransferase; ALAT, alanine aminotransferase.

Data are shown as mean ± sem (*n* 3 cages).

Statistical differences are determined using a one-way ANOVA followed by Tukey’s *post hoc* test.

### Histopathological evaluation

**Fig. 6.**
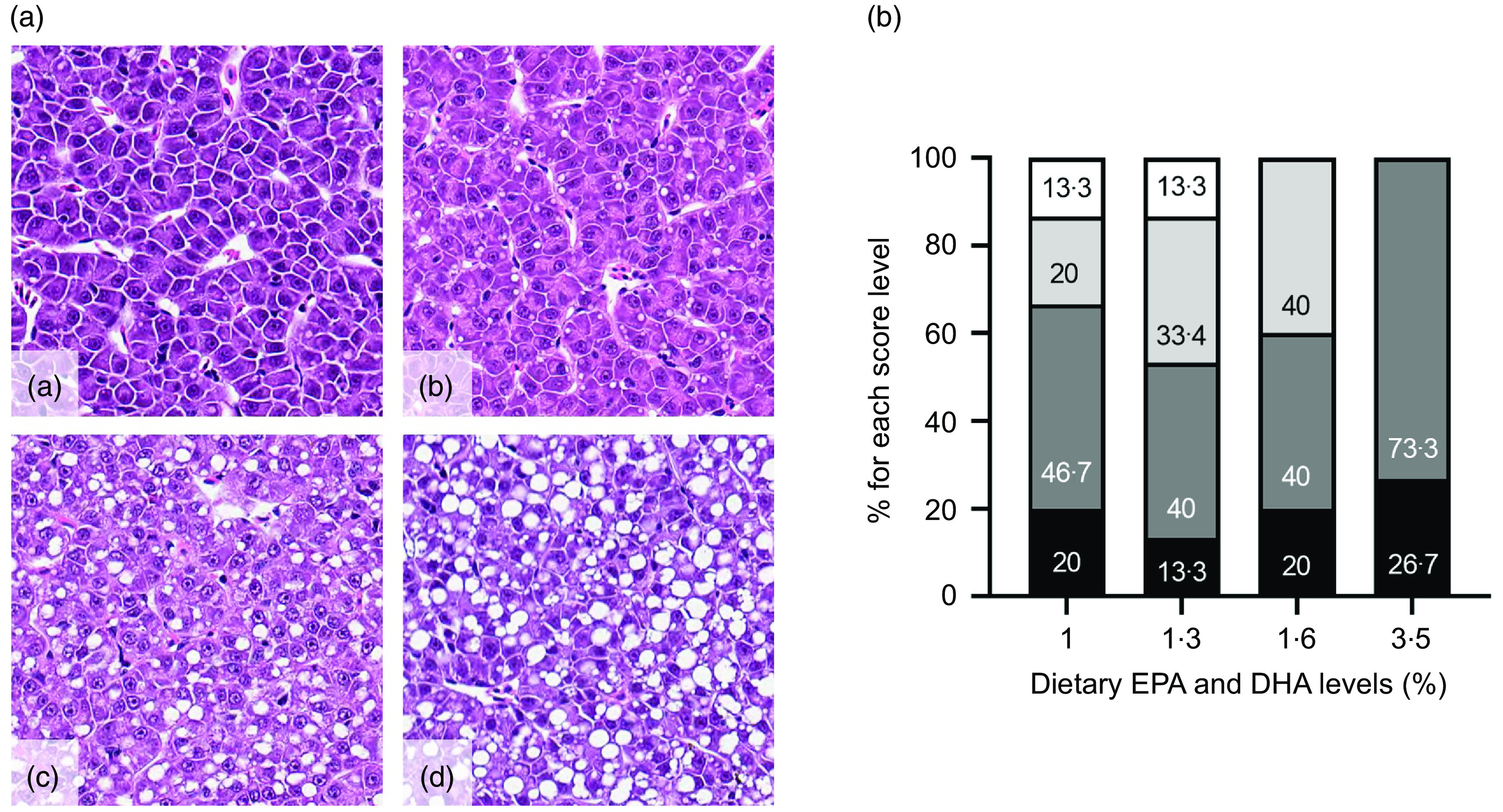
Details of hepatic tissue, representing different levels of lipid vacuoles and customised scoring system (a). Lowercase letters indicate sparse vacuolisation, score 1 (a); moderate but distinct presence of lipid vacuoles, score 2 (b); abundant vacuolisation, score 3 (c) and large lipid vacuoles dominate the tissue, but hepatocytes and other tissue structures appear normal, score 4 (d). Scoring of lipid vacuolisation (1–4) represented as percentage of fish (fifteen fish per group) displaying each score (b). (b) 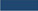, 4; 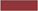, 3; 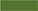, 2; 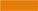, 1/

Histological examination of the liver revealed no signs of specific pathologies, and the overall structure and morphology of the tissues were considered normal. There was variation in the number and size of clear lipid vacuoles within hepatocytes between individual fish, but these were uniform within each section from the same fish. Fig. 6(a) shows representative pictures of the different levels of lipid vacuoles and the customised scoring system. None of the samples was given a score of 5. The average score for lipid vacuolisation was 2·3, 2·5 and 2·2 for the 1·0, 1·3 and 1·6 % EPA and DHA diets, respectively. The average score was 1·7 for the 3·5 % diet. None of the sections from the 3·5 % EPA and DHA group scored >2. In contrast, two livers in each of the 1·0 and 1·3 % EPA and DHA groups were given a score of 4 (Fig. 6(b)).

**Fig. 7.**
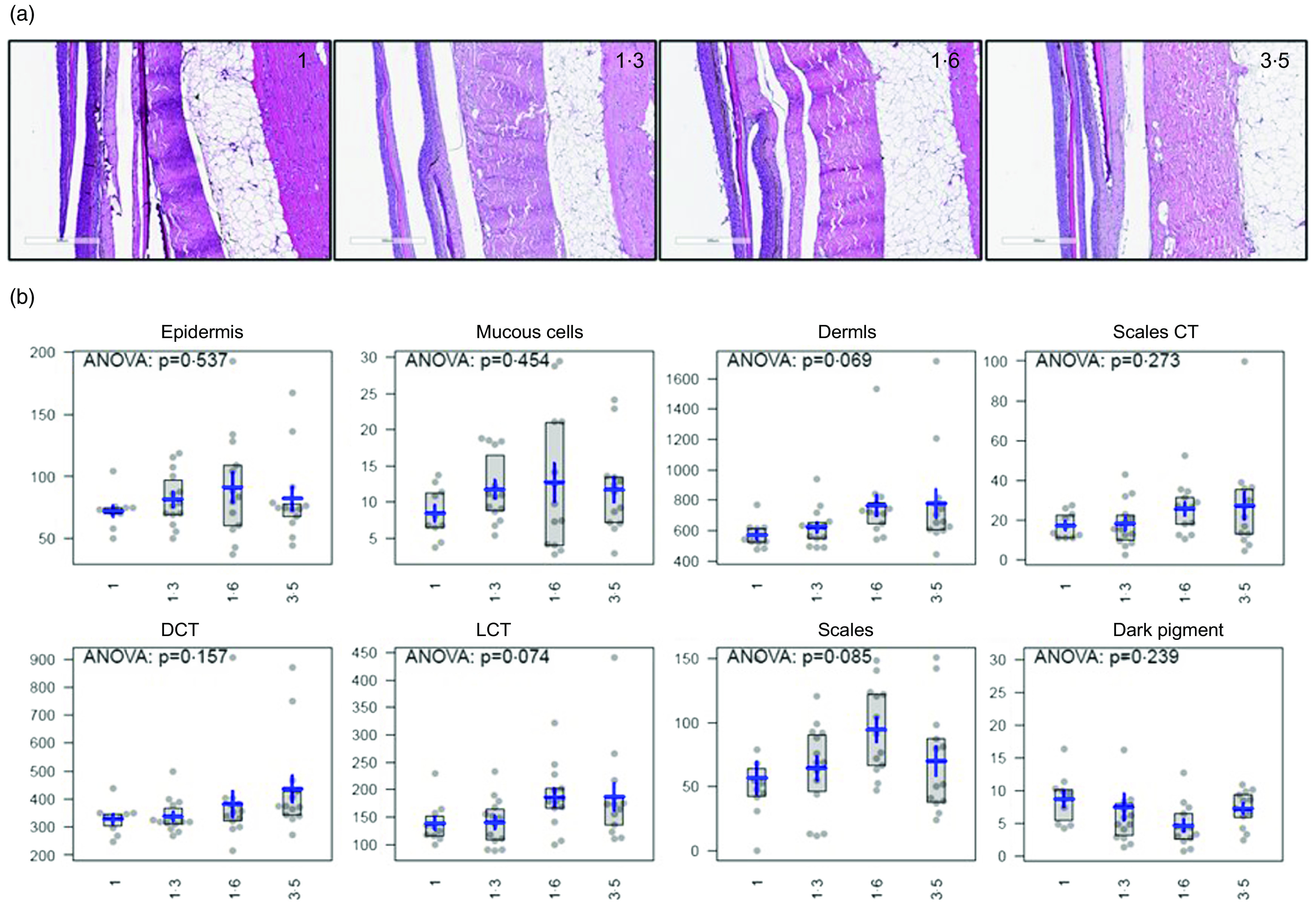
Representative images (a) and analyses of several skin-related traits (b) on skin sections from Atlantic salmon fed the four experimental diets (1, 1·3, 1·6 or 3·5 % EPA and DHA). Areas of epidermis, mucous cells, dermis, scales CT (connective tissue), DCT (dense connective tissue), LCT (loose connective tissue), scales and dark pigment are shown. Units of the *y*-axes are 1000 µm^2^/mm skin. Grey boxes indicate the 2nd and 3rd quartiles (central 50 % of the values). The blue horizontal lines indicate the mean values and blue vertical lines ± sem.

Sections from the middle intestine generally displayed normal tissues throughout all the dietary groups (online Supplementary Fig. S1). Some minor signs of excess vacuolisation in the supranuclear area of enterocytes^(14)^ were observed in two out of fifteen sections in the 1·0 % EPA and DHA group, and in one of the sections from the 1·6 % EPA and DHA group. Furthermore, all fish had a normal outline of distal intestine, with well-developed mucosal folds and highly vacuolised mucosal lining (online Supplementary Fig. S2). Some individual fish had a deviant appearance of the intestinal mucosa, displaying either a very sparse vacuolisation or a foamy appearance of the supranuclear cytoplasm. Reduced vacuolisation was recorded in four fish: one in the 1·0 % EPA and DHA group and three in the 1·3 % EPA and DHA group. A foamy cytoplasm was observed in two fish: one in the 1·0 % EPA and DHA group and one in the 1·6 % EPA and DHA group. In the 3·5 % EPA and DHA group, all samples were considered normal. Furthermore, skin analysis showed no specific pathologies in any of the groups, and all structures evaluated were considered normal. Area and thickness of several skin-related traits, including epidermis, mucous cells, dermis, scales connective tissue, dense connective tissue, loose connective tissue, scales and dark pigment, were evaluated (Fig. 7). No differences between groups for any of the skin components were analysed. However, higher values were observed in the dermis, and a positive tendency towards higher values in dense connective tissues was observed with increasing dietary EPA and DHA, although this was not statistically significant. Interestingly, for some of the skin components evaluated (epidermis, mucous cells, loose connective tissue and scales), the 1·6 % EPA and DHA group displayed the highest values, suggesting that increasing the amount of EPA and DHA in the diets to 3·5 % would not have a further effect on these traits. Furthermore, X-ray analyses of the vertebral columns showed a prevalence of fusions ranging between 16 and 21 % in the various dietary groups (online Supplementary Fig. S3). In addition to fusions, there was a low prevalence of fish with cross-stitch vertebrae (online Supplementary Fig. S3C) in the material. However, no statistical differences were seen among the dietary groups.

### Welfare evaluation and fillet quality

**Fig. 8.**
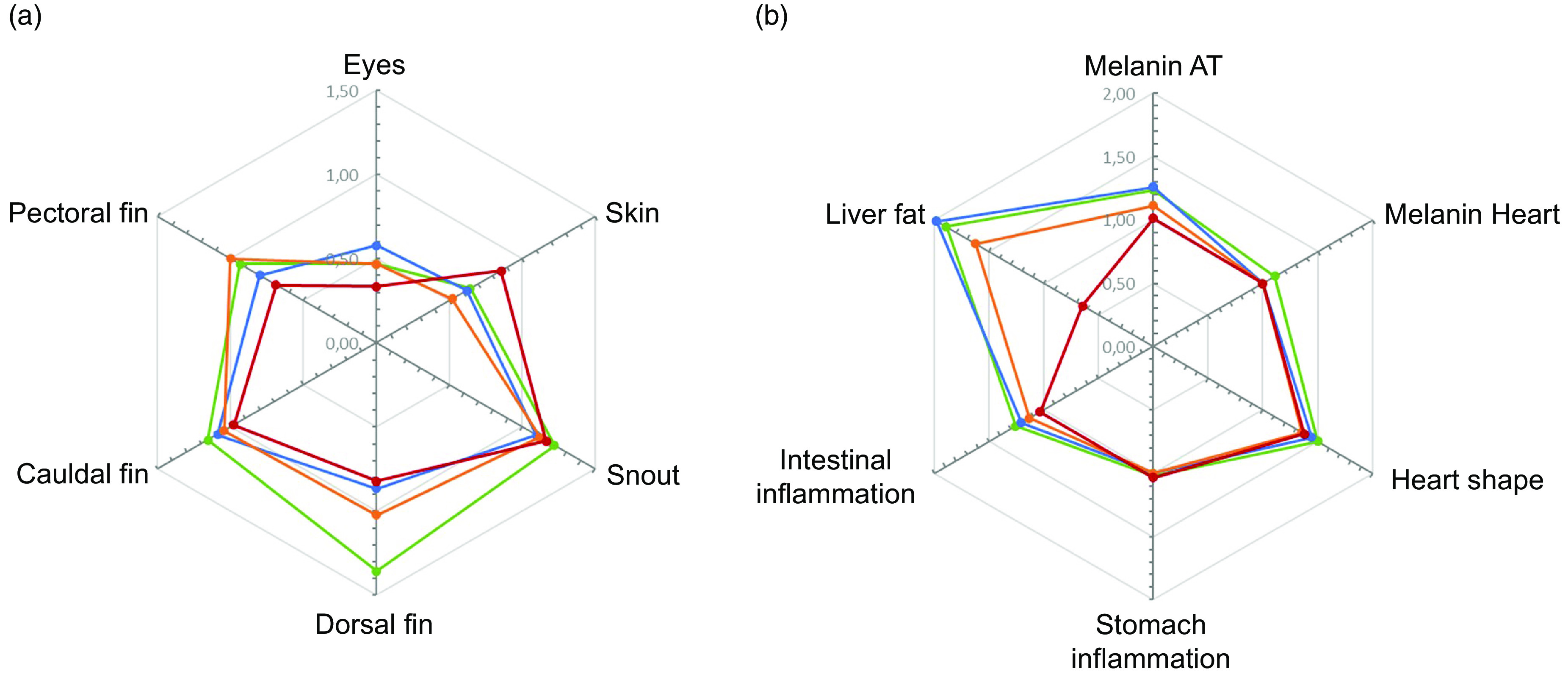
Radar plots representing external (a; eyes, skin, snout, dorsal fin, caudal fin and pectoral fin) and internal (b; melanin adipose tissue, melanin heart, heart shape, stomach inflammation, intestine inflammation and liver fat) welfare indicators of Atlantic salmon fed the four experimental diets (1, 1·3, 1·6 or 3·5 % EPA and DHA). 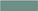, 1 %; 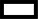, 1·3 %; 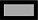, 1·6 %; 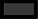, 3·5 %.

External welfare indicators (eye cataract, skin lesions, snout damage and fin damage, including dorsal, pectoral and caudal fins) showed no statistical differences among the groups (Fig. 8(a)); however, the 3·5 % EPA and DHA group showed the lowest occurrence for most indicators except skin damage. In addition, when taken together as an external welfare index, there was reduced welfare in fish fed the 1 % diet (online Supplementary Table S11). On the other hand, among internal organ health indicators (melanin spots in visceral adipose tissue and heart, heart shape, stomach and intestinal inflammation and fat accumulation in the liver), fish fed the 3·5 % EPA and DHA diet showed lower scorings for both melanisation in heart and liver colour (Fig. 8(b)), suggesting improved welfare as indicated by the internal welfare index (online Supplementary Table S11). Fillet astaxanthin and idoxanthin levels, colour, number of melanin spots and liquid loss after freezing and thawing are shown in Table 8. No statistically significant differences were seen for astaxanthin and idoxanthin levels in the fillet. Fillet colour increased with EPA and DHA content in the diets and was significantly higher in the fish fed the 3·5 % EPA and DHA diet compared with all other groups. On the other hand, the prevalence of melanin spots showed a tendency to decrease with increased dietary EPA and DHA content and was significantly decreased in the 3·5 % group compared with the 1 % group. Liquid loss was also reduced significantly as the percentage EPA and DHA in the diet increased, particularly in the 3·5 % EPA and DHA group compared with all the other dietary groups.

**Table 8.** Selected fillet quality parameters of samples from Atlantic salmon fed the four experimental diets (1, 1·3, 1·6 or 3·5 % EPA and DHA) (Mean values with their standard errors)

	Dietary EPA + DHA levels (%)								*P* (ANOVA)
	1·0		1·3		1·6		3·5		
	Mean	sem	Mean	sem	Mean	sem	Mean	sem	
Astaxanthin (mg/kg)	3·9	0·37	4·8	0·65	4·4	0·17	4·6	0·32	0·5353
Idoxanthin (mg/kg)	0·1	0·02	0·2	0·02	0·2	0·01	0·2	0·03	0·1009
Colour (SalmoFan)	23·6^a^	0·14	24·0^a,b^	0·12	24·3^b^	0·14	25·0^c^	0·11	<0·0001
Melanin spots (%)*	35·3^a^	8·32	19·4^a,b^	6·69	20·6^a,b^	7·04	8·6^b^	4·80	0·0484
Liquid loss (%)**	4·1^a^	0·11	4·2^a^	0·18	3·5^a^	0·16	2·8^b^	0·16	<0·0001
EPA + DHA (mg/g)	6·0^a^	0·10	7·4^b^	0·24	8·9^c^	0·24	16·3^d^	0·50	<0·0001

Data are shown as mean ± sem (*n* 3 cages for astaxanthin, idoxanthin (mg/kg fillet) and EPA + DHA levels (mg/g fillet); *n* 43–45 for colour, melanin spots and liquid loss).

Different letters within each row indicate significant differences between values determined using one-way ANOVA followed by Tukey’s *post hoc* test.

*Melanin spots show the percentage of fish with melanin discolouration.

**Liquid loss refers to the weight loss after freezing and thawing (g/100 g fillet).

## Discussion

In recent years, many studies have aimed to determine the minimum dietary requirement of the *n*-3 PUFA, EPA and DHA in Atlantic salmon. It has become clear that, even if acceptable growth can be achieved, using low levels of these PUFA in salmon diets may have a negative impact on nutritional value as well as overall fish health and welfare. Several studies have demonstrated that the FA requirements for optimal growth in Atlantic salmon can be met using approximately 0·5 % (freshwater) and 1 % (seawater) EPA and DHA in the diet^(12,19,29,30)^. Nevertheless, essential FA deficiencies may not always be manifested by reduced growth^(14,15)^, highlighting the existence of more than one requirement level of these essential FA^(10,13,31,32)^. In other words, the requirements to support fish robustness (level 1) may be different than those to ensure adequate growth (level 2) or improve the nutritional quality of fish for consumers (level 3). This is particularly important in aquaculture commercial facilities where fish may face challenging environmental conditions. Unfortunately, there is not enough evidence on the effects of increasing EPA and DHA in salmon diets above the current commercial levels due to the need to reduce the use of marine ingredients rich in these essential FA. Therefore, it is necessary to re-evaluate the potential benefits of increasing EPA and DHA levels in salmon diets. The present study aimed to test different dietary EPA and DHA levels ranging from low (1·0 %, previously defined as minimum) and medium (1·3 and 1·6 %) to high (3·5 %) in Atlantic salmon reared through a whole cycle in sea cages, with a special focus on growth, health, welfare and fillet quality.

Based on observations from studies with a controlled production environment as well as short-term trials, some authors have suggested that the current commercial aquafeed formulations for salmon already include a physiological excess of EPA and DHA and that higher dietary levels do not significantly improve growth performance^(34,35)^. It is important to consider the differences in experimental designs (fish size, life stage, trial duration, etc.), which are essential determinants when assessing *n*-3 PUFA requirements. In the present study, fish fed the diet containing the highest amounts of EPA and DHA (3·5 %) displayed a significant increase in growth in terms of TGC (approximately 5 %), even compared with those fed the 1·6 % EPA and DHA diet, which was previously defined as the required level to ensure optimal growth^(15,29,33)^. However, these differences were only found towards the end of the experiment, indicating that long-term feeding trials are required to determine FA requirements for optimal growth as previously reported^(19)^. One possible explanation for the differences in growth found at the end of our trial could be related to stress induced by a delousing procedure (August 2018) as well as the onset of a CMS outbreak (September 2018). It has been extensively demonstrated that dietary and environmental challenges influence fish health and can ultimately have a massive impact on growth performance^(36)^, particularly when stress and diseases come into play^(37–39)^. In addition, although no significant differences were found in feed intake for the whole feeding trial period, fish fed the 3·5 % EPA and DHA diet, when compared with the other groups, had a higher SFR and showed a tendency to increased feed intake during the last part of the trial suggesting an improved feed utilisation during a stressful period (delousing and CMS outbreak). Nevertheless, it is important to note that the CMS outbreak was naturally occurring and that the main purpose of the present study was not to test effects of dietary treatments against this disease. Therefore, previously described preventive measures, including reduced feeding and handling, were adopted during CMS outbreak in order to reduce stress and improve the recovery of the experimental fish^(40)^.

Several studies have reported that the requirements for *n*-3 PUFA in fish are relative to dietary lipid level^(13,41,42)^ and should be 5–8 % of the total FA, which is the proposed required level for optimal growth in salmon^(43)^. It should be noted that although there was no difference in dietary lipid levels between groups within each period in our trial, the lipid level varied between the different feed productions depending on the fish size (from 30 % at the beginning to 40 % lipid at the end of the trial) and thereby reduced the percentages of EPA and DHA relative to total FA with increasing fat percentages in the diets. In the present study, only the 3·5 % EPA and DHA diet was above these levels (approximately 10 % of the total FA) for all the feed productions, while the other diets contained approximately 5 % of total FA (1·3 and 1·6 % EPA and DHA diets) or less (1 % EPA and DHA diet), which gradually decreased as the dietary lipid levels increased during the trial. In this regard, the lower growth observed in the 1, 1·3 and 1·6 % dietary treatments may thus suggest that only the 3·5 % group had the requirements of EPA and DHA covered throughout the experiment. It is important to note that due to practical reasons, the 3·5 % diet was formulated with more FM, rich in marine phospholipids, as well as vitamin and mineral premixes in the first feed production (October 2017 to January 2018). Nevertheless, the measured EPA and DHA levels of the 3·5 % diets in this and the second feed production (January 2018 to April 2018) were a bit lower compared with the other productions as reported in Table 1, which could potentially explain the lack of growth differences at earlier sampling points. These slight differences in the amount of FM, vitamins and minerals as well as between targeted and measured EPA and DHA levels in these feeds used at the beginning of the trial most likely had a minimal influence on the results at the final sampling. Taken together, our data show that increasing the dietary levels of EPA and DHA to 3·5 % may be a good strategy to improve growth, highlighting the relevance of conducting long-term experiments in fish reared in sea cages that mimic the potential challenges that the fish may encounter in standard commercial facilities.

As expected, the FA composition of the whole body and the different tissues analysed generally reflected the composition of the respective diets. EPA and DHA levels showed linear relationships with dietary EPA and DHA, as observed previously^(15,44,45)^; however, slight differences were found depending on the tissue. Muscle (fillet) and skin showed almost exactly the same contents of EPA and DHA, as previously reported^(15)^. Particularly, EPA and DHA levels in fillet of the 3·5 % group were higher (1600 mg/100 g) than the average commercial levels (1220 mg/100 g) reported by the Norwegian Institute of Marine Research in 2020^(46)^, suggesting an increased nutritional quality. Furthermore, liver showed a particularly higher increase in EPA and DHA as compared with the other tissues. Whole body and tissue fat content showed no significant differences, except liver, which showed a concomitant reduction in fat content with the increase in EPA and DHA as previously reported^(47)^, suggesting a potential health benefit as excessive fat deposition is a recognised health problem in farmed salmon^(48)^. This is in agreement with the lower incidence of abundant vacuolisation found in the histopathological evaluation of the liver from the 3·5 % EPA and DHA group. The EPA and DHA levels in the middle intestine also appeared to increase following the dietary levels but less apparent compared with the other tissues, while the distal intestine showed almost no linear correspondence to the dietary FA content. The FA composition in the middle intestine usually reflects the composition of the diets as seen in the other tissues^(15)^. Intestinal health is essential for both growth and disease resistance, since malfunction of these barrier tissues can impair nutrient uptake and cause inflammatory disorders which may increase the susceptibility to eventual environmental stressors^(49,50)^. The results on the distal intestine suggest that the parts of the intestine that are mostly involved in the absorption of FA, pyloric caeca and middle intestine are more influenced by the dietary FA than the distal intestine where little absorption of dietary FA takes place.

Furthermore, the retention of *n*-3 PUFA, particularly EPA and DHA, varied in the different periods analysed. During the first period (fish approximately 600 g in weight), EPA and DHA showed a parallel deposition with the increase in dietary EPA and DHA. In contrast, this trend reversed during the other two periods, with lower deposition of EPA and especially DHA as they increased in the diet. Generally, EPA and DHA retention decreases when there is high inclusion of these PUFA in the diet^(34,44,51)^. This reduced efficiency at increased dietary levels is arguably produced due to feedback inhibition of the endogenous fatty acyl desaturases by EPA and particularly DHA^(52,53)^. However, in agreement with the results obtained during this first period, some studies have reported high *n*-3 PUFA retention in whole body and muscle when feeding high levels of EPA and DHA^(33,43,54,55)^. Once again, the contrasting results seen in different studies may be attributed to different experimental designs. A relationship between *n*-3 PUFA retention and seasonal variations, particularly related to temperature fluctuation, has been proposed^(56)^. In this regard, during autumn and winter, when the water temperature substantially decreases, fish tend to deposit more FA, possibly as a reflection of thermal adaptation mechanisms, whereas there is a decrease in FA retention when the temperature rises again in spring and summer, which may be redirected to fulfil energy demands.

EPA and DHA requirements should also be determined based on fish health and welfare. In this regard, apart from the nutritional value of high *n*-3 PUFA deposition in fish fillets, our study showed that increasing the dietary EPA and DHA content to 3·5 % has a positive impact on salmon health and welfare. Although no significant differences in mortality rates were observed over the course of the trial, a clear tendency was observed during the CMS outbreak. In particular, fish fed the 3·5 % EPA and DHA diet showed the lowest number of dead fish due to this disease (confirmed by an experienced veterinarian), suggesting that high levels of dietary EPA and DHA improve fish robustness, which was also reported previously^(14)^. In contrast, previous studies found that higher levels of these PUFA in the diet did not result in lower mortality rates^(19)^, even in the presence of diseases such as CMS or pancreatic disease^(16)^. Our findings are in agreement with the tendency towards higher plasma CK values with lower levels of dietary EPA and DHA, which is a common sign for myocardial dysfunction diagnosis, although our findings were not statistically significant^(57,58)^. Nonetheless, both histoscoring and gene expression analyses (not shown) from the atria and ventricles of the heart showed no significant differences among the dietary groups. In this regard, it is important to note that, although a tendency towards lower mortality was observed during this period, the present study was not designed to test the efficiency of the dietary treatments against a CMS infection, as previously mentioned, and that additional information from more sampling points would be necessary to assess whether the 3·5 % group would have a significant advantage at different stages of the disease.

Histological examination showed no specific pathologies in any of the evaluated tissues; however, analysis revealed a tendency towards healthier tissues as the amount of EPA and DHA in the diet increased, although this was not statistically significant. Fish fed the 3·5 % EPA and DHA diet showed a lower degree of lipid vacuolisation in the liver compared with fish fed the other diets, which is in agreement with the lower lipid content in this tissue. Skin analyses also showed a similar trend in several skin-related traits, although it stopped at 1·6 % EPA and DHA, suggesting that higher dietary levels of EPA and DHA would not induce further beneficial effects. However, it is worth noting that the fish in the present study were fed the experimental diets relatively late after smoltification compared with other feeding trials that commenced in the freshwater phase^(14,15)^. Hence, a further negative impact of low dietary EPA and DHA inclusion (1·0 %), especially in histopathological analyses, could have been observed in tissues such as liver and intestine, as previously reported^(14)^. In line with these results, analysis of internal organ health scores and external welfare indicators revealed the same tendency towards improved welfare with increased dietary EPA and DHA content, especially for liver colour, which is considered a visual indicative of fat accumulation^(59)^ and is therefore associated with nutritional disorders^(60)^. On the other hand, the welfare scorings for skin damage appeared to be marginally higher (worse condition) but not significant in the 3·5 % EPA and DHA group, which was unexpected given the demonstrated positive impact of *n*-3 PUFA in barrier tissues, such as skin^(61)^. Although only anecdotical, one potential explanation for these results could be that the fish in the 3·5 % EPA and DHA group were substantially bigger and more active at the time of the final sampling and handling may have slightly damaged the skin and had a negative influence on the scoring.

One of the main goals of the Atlantic salmon industry is the production of high-quality food, which comprises not only the nutritional value in terms of *n*-3 PUFA levels but also the attractive visual appearance of the fillets^(62,63)^. Therefore, sufficient fillet colour intensity is important for customer acceptance as well as the absence of dark discoloured spots, which currently are a major cause of quality downgrading of farmed salmon fillets^(25)^. Dietary supplementation of carotenoids, mainly astaxanthin, is most commonly used for pigmentation of salmonid fish and its retention in tissues is influenced by the nutritional and physiological status of the fish^(64,65)^. Our results showed significantly redder fillet colour with increasing dietary EPA and DHA levels, but only salmon fed 3·5 % EPA and DHA had an acceptable visual colour of SalmoFan score 25^(63)^. The unacceptable low colour score of the salmon fed 1·0–1·6 % EPA and DHA supports the claim that pale fillet colour has become a serious quality problem during recent years^(66)^. However, no differences in astaxanthin or idoxanthin, an intermediary metabolite of astaxanthin, were observed in the muscle. These results are in agreement with the findings of a recent study that reported improved fillet colour, but not astaxanthin concentration, in salmon fed krill meal rich in EPA and DHA^(59)^. The authors suggested that dietary effects on muscle structure altered the perceived colour appearance. The present study also found an increased liquid holding capacity following freezing and thawing towards the 3·5 % group potentially as a result of higher levels of SFA which increase melting point in tissues as previously reported^(67)^. Moreover, the development of dark discoloured spots has been identified as mild chronic inflammation^(68)^. Our observation of reduced incidence of dark spots with increasing percentage of dietary EPA and DHA coincides with results reported by Sissener *et al.* ^(16)^, who hypothesised that high *n*-3 diets have a positive effect on inflammatory processes in salmon.

Despite several constrains, such as limited supply and increasingly high cost, FO remains the most reliable and economic source of *n*-3 long-chain PUFA^(30)^. However, due to the projected increased aquaculture production (approximately 14 %) leading to increased demand for *n*-3 long-chain PUFA, alternative new sources that are rich in EPA and DHA will be essential to meet the ‘gap’ between supply and demand. Several studies have explored the potential use of other ingredients such as microalgae^(69,70)^ or GM plant oils^(71)^, which would support the use of higher dietary levels of EPA and DHA in Atlantic salmon.

### Conclusions

Although the present feeding trial was not designed to determine dietary EPA and DHA deficiencies below a given threshold, our findings suggest that the levels of these essential FA in Atlantic salmon should be reassessed and that the optimal requirements in terms of fish robustness, growth and quality are higher than those previously reported. In contrast with previous assumptions, our findings indicate that increasing EPA and DHA levels to 3·5 % of the diet significantly increases weight and growth rates under challenging experimental conditions, shows a tendency towards reduced mortality related to stressful environmental conditions and improves the overall fillet quality in Atlantic salmon reared in sea cages. Nevertheless, further studies are required to evaluate the potential optimal inclusion levels of dietary EPA and DHA between 1·6 and 3·5 %, which may appear as a relatively high range in terms of beneficial effects/feed cost.

## Supplementary material

For supplementary material accompanying this paper visit https://doi.org/10.1017/S0007114522000642. click here to view supplementary material
