# Supplementary material for: Increasing dietary levels of the n-3 long-chain PUFA, EPA and DHA, improves the growth, welfare, robustness and fillet quality of Atlantic salmon in sea cages
Source: Br J Nutr. 2022 Mar 3;129(1):10–28. doi: 10.1017/S0007114522000642 (PMC9816656; doi:10.1017/S0007114522000642)
Supplement: Supplementary file 1 [file S0007114522000642sup001.docx]

**Supplementary tables**

**Table S1. Formulation and chemical composition of the experimental diets (****4-mm pellet; fish weight ~200 g; October 2017 to January 2018).**

|  | **Dietary EPA + DHA levels (%)** | | | |
| --- | --- | --- | --- | --- |
| **Formulation^†^** | **1.0** | **1.3** | **1.6** | **3.5** |
| Rapeseed oil, crude^1^ | 21.6 | 20.1 | 18.6 | 8.7 |
| Soya protein concentrate | 22.8 | 25.4 | 26.4 | - |
| Wheat^1^ | 13.1 | 11.3 | 11.3 | - |
| Fish oil^2^ | 2.3 | 4.8 | 6.3 | 9.5 |
| Wheat gluten^3^ | 12.1 | 9.6 | 9.1 | - |
| Fish meal^2^ | 15.0 | 15.0 | 15.0 | 41.5 |
| Maize gluten^4^ | 5 | 5 | 5 | - |
| Pea protein^5^ | 4 | 5 | 5 | - |
| Monocalcium phosphate | 1.5 | 1.5 | 1.4 | - |
| Vitamin and mineral premix^6^ | 0.8 | 0.8 | 0.8 | 1.8 |
| Amino acids^7^ | 2.0 | 2.0 | 1.8 | 0.6 |
| Lucatin Pink CWD, 10% | 0.06 | 0.06 | 0.06 | - |
| Lucatin Pink CWD, 20% | - | - | - | 0.001 |
| Water change | -0.8 | -0.7 | -0.7 | -0.2 |
| **Chemical composition** |  |  |  |  |
| Moisture (%) | 6 | 6 | 6 | 6 |
| Energy, crude (MJ/kg) | 23.1 | 23.2 | 23.3 | 23.6 |
| Protein, crude (%) | 42.9 | 43.2 | 43.0 | 45.2 |
| Fat, crude (%) | 27.9 | 28.3 | 28.3 | 27.7 |
| Ash (%) | 6.2 | 6.4 | 6.3 | 8.4 |

^†^g/100 g feed

^1^Denmark; ^2^Peru/Denmark; ^3^EU; ^4^Ukraine; ^5^China; ^6^Sweden; ^7^Germany/Korea/China

**Table S2. Formulation and chemical composition of the experimental diets (****6-mm pellet; fish weight ~500 g; January 2018 to April 2018).**

|  | **Dietary EPA + DHA levels (%)** | | | |
| --- | --- | --- | --- | --- |
| **Formulation^†^** | **1.0** | **1.3** | **1.6** | **3.5** |
| Rapeseed oil, crude^1^ | 24.1 | 22.1 | 20.7 | 15.6 |
| Soya protein concentrate | 30 | 30 | 30 | 30 |
| Wheat^1^ | 11.3 | 11.3 | 11.3 | 11.3 |
| Fish oil^2^ | 3.4 | 5.4 | 6.8 | 11.8 |
| Wheat gluten^3^ | 8.9 | 9.6 | 9.6 | 5.8 |
| Fish meal^2^ | 11 | 10 | 10 | 10 |
| Maize gluten^4^ | 5 | 5 | 5 | 5 |
| Pea protein^5^ | - | - | - | 5 |
| Sunflower expeller | 3.0 | 3.0 | 3.1 | 2.6 |
| Monocalcium phosphate | 1.3 | 1.4 | 1.4 | 1.4 |
| Vitamin and mineral premix^6^ | 0.9 | 0.9 | 0.9 | 0.9 |
| Amino acids^7^ | 1.6 | 1.7 | 1.7 | 1.4 |
| Lucatin Pink CWD, 10% | 0.06 | 0.06 | 0.06 | 0.06 |
| Water change | -0.47 | -0.43 | -0.44 | -0.58 |
| **Chemical composition** |  |  |  |  |
| Moisture (%) | 6 | 6 | 6 | 6 |
| Energy, crude (MJ/kg) | 24.5 | 24.5 | 24.5 | 24.5 |
| Protein, crude (%) | 39.5 | 39.5 | 39.4 | 39.5 |
| Fat, crude (%) | 30.4 | 30.4 | 30.4 | 30.4 |
| Ash (%) | 5.8 | 5.8 | 5.7 | 5.7 |

^†^g/100 g feed

^1^Denmark; ^2^Peru/Denmark; ^3^EU; ^4^Ukraine; ^5^China; ^6^Sweden; ^7^Germany/Korea/China

**Table S3. Formulation and chemical composition of the experimental diets (****9-mm pellet; fish weight ~1,000 g; April 2018 to August 2018).**

|  | **Dietary EPA + DHA levels (%)** | | | |
| --- | --- | --- | --- | --- |
| **Formulation^†^** | **1.0** | **1.3** | **1.6** | **3.5** |
| Rapeseed oil, crude^1^ | 25.9 | 24.3 | 22.8 | 14.9 |
| Soya protein concentrate | 11.4 | 11.4 | 11.4 | 9.8 |
| Guar meal^2^ | 20 | 20 | 20 | 20 |
| Wheat^1^ | 10.8 | 10.8 | 10.8 | 11.5 |
| Fish oil^3^ | 4.3 | 5.8 | 7.3 | 15 |
| Wheat gluten^4^ | 2.2 | 2.2 | 2.2 | 2.8 |
| Fish meal^3^ | 5 | 5 | 5 | 5 |
| Maize gluten^5^ | 5 | 5 | 5 | 5 |
| Vitamin and mineral premix^6^ | 2.9 | 2.9 | 2.9 | 2.9 |
| Amino acids^7^ | 1.3 | 1.3 | 1.3 | 1.4 |
| Lucatin Pink CWD, 10% | 0.06 | 0.06 | 0.06 | 0.06 |
| Water change | 0.5 | 0.5 | 0.5 | 0.5 |
| **Chemical composition** |  |  |  |  |
| Moisture (%) | 6 | 6 | 6 | 6 |
| Energy, crude (MJ/kg) | 25.3 | 25.3 | 25.3 | 25.3 |
| Protein, crude (%) | 36.2 | 36.2 | 36.2 | 36.2 |
| Fat, crude (%) | 34.7 | 34.7 | 34.7 | 34.6 |
| Ash (%) | 5.5 | 5.5 | 5.5 | 5.5 |

^†^g/100g feed

^1^Denmark; ^2^India; ^3^Peru/Denmark; ^4^EU; ^5^Ukraine; ^6^Sweden; ^7^Germany/Korea/China

**Table S4. Formulation and chemical composition of the experimental diets (****9-mm pellet; fish weight ~2,500 g; August 2018 to September 2018).**

|  | **Dietary EPA + DHA levels (%)** | | | |
| --- | --- | --- | --- | --- |
| **Formulation^†^** | **1.0** | **1.3** | **1.6** | **3.5** |
| Rapeseed oil, crude^1^ | 30.6 | 29.1 | 27.8 | 19.6 |
| Soya protein concentrate | 11.0 | 8.4 | 8.4 | 3.1 |
| Guar meal | 17.6 | 20.0 | 20.0 | 20.0 |
| Wheat^1^ | 10.9 | 10.8 | 10.8 | 13.8 |
| Fish oil^2^ | 3.8 | 5.1 | 6.4 | 14.2 |
| Wheat gluten^3^ | 6.9 | 7.0 | 6.9 | 9.4 |
| Fish meal^2^ | 5 | 5 | 5 | 5 |
| Maize gluten^4^ | 5 | 5 | 5 | 5 |
| Pea protein^5^ | 5 | 5 | 5 | 6 |
| Monocalcium phosphate | 1.3 | 1.3 | 1.3 | 1.3 |
| Vitamin and mineral premix^6^ | 1.7 | 1.7 | 1.7 | 1.7 |
| Amino acids^7^ | 1.0 | 1.1 | 1.1 | 1.3 |
| Lucatin Pink CWD, 20% | 0.03 | 0.03 | 0.03 | 0.03 |
| Water change | 0.5 | 0.6 | 0.6 | 0.6 |
| **Chemical composition** |  |  |  |  |
| Moisture (%) | 6 | 6 | 6 | 6 |
| Energy, crude (MJ/kg) | 26.1 | 26.1 | 26.1 | 26.0 |
| Protein, crude (%) | 33.5 | 33.6 | 33.6 | 33.5 |
| Fat, crude (%) | 38.2 | 38.2 | 38.2 | 37.7 |
| Ash (%) | 4.5 | 4.5 | 4.5 | 4.3 |

^†^g/100 g feed

^1^Denmark; ^2^Peru/Denmark; ^3^EU; ^4^Ukraine; ^5^China; ^6^Sweden; ^7^Germany/Korea/China

| Melanin adipose tissue |  | |  | |  |  |  |  |  |  |  |
| --- | --- | --- | --- | --- | --- | --- | --- | --- | --- | --- | --- |
|  | | Score 1 | | Clear white fat | | |  |  |  |  |  |
|  | | Score 2 | | Whitish, few dark spots | | | |  |  |  |  |
|  | | Score 3 | | Pale grey, many dark spots | | | |  |  |  |  |
| Melanin Heart | |  | |  | |  |  |  |  |  |  |
|  | | Score 1 | | No fat deposits with melanin | | | |  |  |  |  |
|  | | Score 2 | | Fat deposits with melanin | | | |  |  |  |  |
| Heart shape | |  | |  | |  |  |  |  |  |  |
|  | | Score 1 | | Normal, deep red, pear-shaped, little fat deposit stands on its point. | | | | | | | |
|  | | Score 2 | | Slight pink, focal fat deposits. | | | |  |  |  |  |
|  | | Score 3 | | Multifocal fat deposits, loss of shape. | | | | |  |  |  |
|  | | Score 4 | | Rounded, multiple fat deposits, small, does not hold shape | | | | | | |  |
| Stomach inflammation | |  | |  | |  |  |  |  |  |  |
|  | | Score 1 | | Normal, pale, | | |  |  |  |  |  |
|  | | Score 2 | | Slight pink, focal patches | | | |  |  |  |  |
|  | | Score 3 | | Multifocal patches | | |  |  |  |  |  |
|  | | Score 4 | | Dark red, diffuse patches | | | |  |  |  |  |

**Table S5. Welfare indicator scoring system developed by Biomar AS, Norway.**

**Table S6. Total lipid content and fatty acid composition (% of total) in the livers of Atlantic salmon fed different experimental diets. Data are shown as mean ± SEM (n = 3 cages).**

|  | **1.0% EPA + DHA** | **1.3% EPA + DHA** | **1.6% EPA + DHA** | **3.5% EPA + DHA** | ***P*-value (ANOVA)** |
| --- | --- | --- | --- | --- | --- |
| **Total fat** | **10.5 ± 0.80^ab^** | **12.4 ± 1.86^a^** | **10.1 ± 0.57^ab^** | **6.0 ± 0.45^b^** | **0.0164** |
| 14:0 | 0.7 ± 0.01^a^ | 0.8 ± 0.02^ab^ | 0.9 ± 0.02^b^ | 1.4 ± 0.05^c^ | <0.0001 |
| 16:0 | 5.9 ± 0.17^a^ | 5.6 ± 0.54^a^ | 5.8 ± 0.08^a^ | 9.9 ± 0.28^b^ | <0.0001 |
| 18:0 | 3.5 ± 0.08^ab^ | 3.6 ± 0.13^ab^ | 3.4 ± 0.12^a^ | 4.5 ± 0.44^b^ | 0.0345 |
| **Σ SFA^1^** | **10.7 ± 0.26^a^** | **10.6 ± 0.76^a^** | **10.7 ± 0.19^a^** | **16.7 ± 0.72^b^** | **<0.0001** |
| 16:1 *n*-7 | 1.1 ± 0.21^a^ | 1.1 ± 0.06^a^ | 1.4 ± 0.10^ab^ | 1.9 ± 0.10^b^ | 0.0084 |
| 18:1 *n*-9 | 44.2 ± 0.77^a^ | 45.0 ± 1.49^a^ | 42.5 ± 0.89^a^ | 26.3 ± 0.61^b^ | <0.0001 |
| 18:1 *n*-7 | 3.0 ± 0.02^a^ | 3.1 ± 0.05^ab^ | 3.2 ± 0.04^b^ | 3.1 ± 0.05^ab^ | 0.0430 |
| 20:1 *n*-9 | 4.4 ± 0.02 | 4.8 ± 0.16 | 4.5 ± 0.13 | 4.2 ± 0.12 | 0.0681 |
| 22:1 *n*-7 | 0.8 ± 0.05 | 0.8 ± 0.06 | 0.8 ± 0.03 | 0.9 ± 0.02 | 0.1413 |
| 22:1 *n*-11 | 0.1 ± 0.00^a^ | 0.1 ± 0.01^a^ | 0.2 ± 0.01^a^ | 0.6 ± 0.07^b^ | <0.0001 |
| **Σ MUFA^2^** | **55.1 ± 0.69^a^** | **56.3 ± 1.52^a^** | **53.7 ± 1.05^a^** | **38.9 ± 0.89^b^** | **<0.0001** |
| 18:2 *n*-6 | 14.0 ± 0.38^a^ | 13.8 ± 0.27^a^ | 13.3 ± 0.15^a^ | 8.4 ± 0.15^b^ | <0.0001 |
| 20:2 *n*-6 | 2.3 ± 0.05^a^ | 2.5 ± 0.05^a^ | 2.5 ± 0.05^a^ | 2.1 ± 0.04^b^ | 0.0009 |
| 20:3 *n*-6 | 1.0 ± 0.00^a^ | 0.8 ± 0.06^b^ | 0.6 ± 0.01^b^ | 0.3 ± 0.02^c^ | <0.0001 |
| 20:4 *n*-6 | 0.9 ± 0.21^ab^ | 0.6 ± 0.10^a^ | 0.8 ± 0.05^ab^ | 1.4 ± 0.16^b^ | 0.0211 |
| 18:3 *n*-3 | 4.3 ± 0.07^a^ | 4.4 ± 0.27^a^ | 4.6 ± 0.10^a^ | 2.7 ± 0.07^b^ | <0.0001 |
| 20:3 *n*-3 | 0.6 ± 0.03 | 0.8 ± 0.06 | 0.7 ± 0.14 | 0.6 ± 0.01 | 0.5055 |
| 20:5 *n*-3 | 3.1 ± 0.24^a^ | 2.8 ± 0.36^a^ | 3.5 ± 0.21^a^ | 7.4 ± 0.36^b^ | <0.0001 |
| 22:5 *n*-3 | 0.9 ± 0.06^a^ | 0.8 ± 0.12^a^ | 1.0 ± 0.09^a^ | 2.5 ± 0.09^b^ | <0.0001 |
| 22:6 *n*-3 | 6.0 ± 0.50^a^ | 5.2 ± 0.71^a^ | 7.0 ± 0.63^a^ | 16.1 ± 0.52^b^ | <0.0001 |
| **EPA + DHA** | **9.0 ± 0.74^a^** | **8.0 ± 1.07^a^** | **10.4 ± 0.84^a^** | **23.5 ± 0.88^b^** | **<0.0001** |
| **Σ PUFA^3^** | **33.2 ± 0.41^a^** | **32.2 ± 0.78^a^** | **34.5 ± 1.07^a^** | **42.3 ± 0.83^b^** | **<0.0001** |
| **Σ *n*-3** | **15.1 ± 0.79^a^** | **14.3 ± 0.88^a^** | **17.1 ± 0.98^a^** | **29.9 ± 0.94^b^** | **<0.0001** |
| **Σ *n*-6** | **18.1 ± 0.37^a^** | **17.9 ± 0.16^a^** | **17.4 ± 0.16^a^** | **12.5 ± 0.11^b^** | **<0.0001** |

^a,b,c,d^ Mean values within a row with unlike superscript letters were significantly different (*P* < 0.05; one-way ANOVA followed by Tukey’s honest significant difference test).

^1^Includes 15:0, 17:0, 20:0, 22:0, 24:0.

^2^Includes 14:1 *n*–5, 16:1 *n*-5, 16:1 *n*-9, 17:1 *n*-7, 18:1 *n*-11, 20:1  *n* -7, 20:1 *n*-11, 22:1 *n*-9, 24:1 *n*-9.

^3^Includes 16:2 *n*-3, 16:2 *n*-6, 16:3 *n*-4, 18:3 *n*-6, 18:4 *n*-3, 20:4 *n*-3.

MUFA, monounsaturated fatty acid; PUFA, polyunsaturated fatty acid; SFA, saturated fatty acid.

**Table S7. Total lipid content and fatty acid composition (% of total) in the skin of Atlantic salmon fed different experimental diets. Data are shown as mean ± SEM (n = 3 cages).**

|  | **1.0% EPA + DHA** | **1.3% EPA + DHA** | **1.6% EPA + DHA** | **3.5% EPA + DHA** | ***P*-value (ANOVA)** |
| --- | --- | --- | --- | --- | --- |
| **Total fat** | **17.3 ± 1.82** | **14.4 ± 1.10** | **17.3 ± 1.78** | **18.1 ± 1.29** | **0.4001** |
| 14:0 | 1.0 ± 0.00^a^ | 1.2 ± 0.01^b^ | 1.4 ± 0.02^c^ | 2.5 ± 0.03^d^ | <0.0001 |
| 16:0 | 8.2 ± 0.20^a^ | 8.5 ± 0.05^ab^ | 8.7 ± 0.09^b^ | 10.6 ± 0.03^c^ | <0.0001 |
| 18:0 | 2.8 ± 0.07 | 2.8 ± 0.22 | 2.7 ± 0.17 | 3.1 ± 0.19 | 0.5604 |
| **Σ SFA^1^** | **12.5 ± 0.21^a^** | **13.5 ± 0.31^a^** | **13.7 ± 0.37^a^** | **17.2 ± 0.19^b^** | <0.0001 |
| 16:1 *n*-7 | 1.2 ± 0.07^a^ | 1.3 ± 0.07^ab^ | 1.6 ± 0.04^b^ | 2.9 ± 0.08^c^ | <0.0001 |
| 18:1 *n*-9 | 46.7 ± 0.20^a^ | 44.4 ± 0.27^b^ | 43.7 ± 0.33^b^ | 35.5 ± 0.13^c^ | <0.0001 |
| 18:1 *n*-7 | 3.0 ± 0.02 | 2.8 ± 0.12 | 3.0 ± 0.02 | 3.1 ± 0.01 | 0.0860 |
| 20:1 *n*-9 | 2.3 ± 0.06 | 2.3 ± 0.05 | 2.3 ± 0.05 | 2.3 ± 0.01 | 0.9632 |
| 22:1 *n*-7 | 0.6 ± 0.02^a^ | 0.7 ± 0.05^ab^ | 0.7 ± 0.01^ab^ | 0.8 ± 0.01^b^ | 0.0288 |
| **Σ MUFA^2^** | **55.9 ± 0.12^a^** | **53.5 ± 0.41^b^** | **53.4 ± 0.44^b^** | **47.4 ± 0.24^c^** | <0.0001 |
| 18:2 *n*-6 | 17.0 ± 0.06^a^ | 16.3 ± 0.08^b^ | 16.0 ± 0.15^b^ | 13.6 ± 0.09^c^ | <0.0001 |
| 20:2 *n*-6 | 1.2 ± 0.07^ab^ | 1.2 ± 0.04^a^ | 1.2 ± 0.02^ab^ | 1.0 ± 0.02^b^ | 0.0406 |
| 20:3 *n*-6 | 0.5 ± 0.01^a^ | 0.4 ± 0.02^b^ | 0.4 ± 0.01^b^ | 0.2 ± 0.00^c^ | <0.0001 |
| 20:4 *n*-6 | 0.3 ± 0.01^a^ | 0.3 ± 0.08^a^ | 0.4 ± 0.02^ab^ | 0.6 ± 0.01^b^ | 0.0207 |
| 18:3 *n*-3 | 6.6 ± 0.02^a^ | 6.4 ± 0.05^b^ | 6.3 ± 0.05^b^ | 5.3 ± 0.02^c^ | <0.0001 |
| 20:3 *n*-3 | 0.4 ± 0.03 | 0.5 ± 0.01 | 0.5 ± 0.04 | 0.4 ± 0.01 | 0.2663 |
| 20:5 *n*-3 | 1.7 ± 0.06^a^ | 2.2 ± 0.02^b^ | 2.5 ± 0.06^c^ | 4.7 ± 0.08^d^ | <0.0001 |
| 22:5 *n*-3 | 0.6 ± 0.02^a^ | 0.7 ± 0.04^a^ | 0.9 ± 0.01^b^ | 1.9 ± 0.04^c^ | <0.0001 |
| 22:6 *n*-3 | 2.0 ± 0.08^a^ | 2.8 ± 0.27^b^ | 3.0 ± 0.12^b^ | 5.2 ± 0.15^c^ | <0.0001 |
| **EPA+DHA** | **3.7 ± 0.14^a^** | **5.0 ± 0.28^b^** | **5.4 ± 0.16^b^** | **10.0 ± 0.23^c^** | <0.0001 |
| **Σ PUFA^3^** | **31.0 ± 0.15^a^** | **31.5 ± 0.30^ab^** | **32.1 ± 0.11^b^** | **33.8 ± 0.27^c^** | <0.0001 |
| **Σ *n*-3** | **11.7 ± 0.14^a^** | **12.8 ± 0.19^b^** | **13.4 ± 0.16^b^** | **18.0 ± 0.20^c^** | <0.0001 |
| **Σ *n*-6** | **19.4 ± 0.01^a^** | **18.7 ± 0.20^b^** | **18.4 ± 0.15^b^** | **15.6 ± 0.15^c^** | <0.0001 |

^a,b,c,d^ Mean values within a row with unlike superscript letters were significantly different (*P* < 0.05; one-way ANOVA followed by Tukey’s honest significant difference test).

^1^Includes 15:0, 17:0, 20:0, 22:0, 24:0.

^2^Includes 14:1 *n*-5, 16:1 *n*-5, 16:1 *n*-9, 17:1 *n*-7, 18:1 *n*-11, 20:1 *-*7, 20:1 *n*-11, 22:1 *n*-9, 24:1 *n*-9.

^3^Includes 16:2 *n*-3, 16:2 *n*-6, 16:3 *n*-4, 18:3 *n*-6, 18:4 *n*-3, 20:4 *n*-3.

MUFA, monounsaturated fatty acid; PUFA, polyunsaturated fatty acid; SFA, saturated fatty acid.

**Table S8. Total lipid content and fatty acid composition (% of total) in the fillet of Atlantic salmon fed the different experimental diets. Data are shown as mean ± SEM (n = 3 cages).**

|  | **1.0% EPA + DHA** | **1.3% EPA + DHA** | **1.6% EPA + DHA** | **3.5% EPA + DHA** | ***P*-value (ANOVA)** |
| --- | --- | --- | --- | --- | --- |
| **Total fat** | **20.4 ± 0.41** | **20.9 ± 1.25** | **20.4 ± 0.72** | **19.9 ± 0.26** | **0.8534** |
| 14:0 | 0.9 ± 0.01^a^ | 1.1 ± 0.01^b^ | 1.4 ± 0.01^c^ | 2.4 ± 0.01^d^ | <0.0001 |
| 16:0 | 7.9 ± 0.10^a^ | 8.3 ± 0.03^b^ | 8.8 ± 0.08^c^ | 10.8 ± 0.09^d^ | <0.0001 |
| 18:0 | 2.3 ± 0.02 | 2.4 ± 0.02 | 2.5 ± 0.01 | 2.6 ± 0.17 | 0.2285 |
| **Σ SFA^1^** | **12.2 ± 0.13^a^** | **12.8 ± 0.02^ab^** | **13.5 ± 0.13^b^** | **16.9 ± 0.32^c^** | **<0.0001** |
| 16:1 *n*-7 | 1.1 ± 0.02^a^ | 1.3 ± 0.02^b^ | 1.6 ± 0.03^c^ | 3.0 ± 0.01^d^ | <0.0001 |
| 18:1 *n*-9 | 47.3 ± 0.15^a^ | 45.9 ± 0.10^b^ | 44.3 ± 0.09^c^ | 35.9 ± 0.10^d^ | <0.0001 |
| 18:1 *n*-7 | 2.8 ± 0.11^a^ | 2.9 ± 0.02^ab^ | 2.9 ± 0.01^ab^ | 3.1 ± 0.00^b^ | 0.0280 |
| 20:1 *n*-9 | 2.3 ± 0.01 | 2.4 ± 0.03 | 2.3 ± 0.03 | 2.3 ± 0.01 | 0.4017 |
| 22:1 *n*-7 | 0.6 ± 0.02^a^ | 0.6 ± 0.00^a^ | 0.7 ± 0.00^a^ | 0.8 ± 0.02^b^ | <0.0001 |
| 22:1 *n*-11 | 0.3 ± 0.00^a^ | 0.3 ± 0.02^b^ | 0.4 ± 0.00^c^ | 0.8 ± 0.01^d^ | <0.0001 |
| **Σ MUFA^2^** | **56.0 ± 0.21^a^** | **55.1 ± 0.10^b^** | **53.8 ± 0.04^c^** | **48.0 ± 0.06^d^** | **<0.0001** |
| 18:2 *n*-6 | 17.4 ± 0.07^a^ | 17.1 ± 0.05^b^ | 16.4 ± 0.04^c^ | 13.8 ± 0.04^d^ | <0.0001 |
| 20:2 *n*-6 | 1.1 ± 0.01^a^ | 1.2 ± 0.02^a^ | 1.1 ± 0.04^a^ | 1.0 ± 0.02^b^ | 0.0015 |
| 20:3 *n*-6 | 0.5 ± 0.01 | 0.4 ± 0.01 | 0.4 ± 0.01 | 0.3 ± 0.08 | 0.0703 |
| 20:4 *n*-6 | 0.2 ± 0.00^a^ | 0.2 ± 0.01^b^ | 0.2 ± 0.00^c^ | 0.4 ± 0.00^d^ | <0.0001 |
| 18:3 *n*-3 | 6.8 ± 0.03^a^ | 6.7 ± 0.05^ab^ | 6.6 ± 0.05^b^ | 5.4 ± 0.01^c^ | <0.0001 |
| 20:3 *n*-3 | 0.4 ± 0.01^ab^ | 0.4 ± 0.01^ab^ | 0.5 ± 0.02^a^ | 0.4 ± 0.01^b^ | 0.0487 |
| 20:5 *n*-3 | 1.5 ± 0.02^a^ | 1.8 ± 0.02^b^ | 2.2 ± 0.01^c^ | 4.3 ± 0.04^d^ | <0.0001 |
| 22:5 *n*-3 | 0.6 ± 0.01^a^ | 0.6 ± 0.11^a^ | 0.9 ± 0.01^b^ | 1.9 ± 0.03^c^ | <0.0001 |
| 22:6 *n*-3 | 2.0 ± 0.02^a^ | 2.3 ± 0.10^b^ | 2.8 ± 0.02^c^ | 5.2 ± 0.03^d^ | <0.0001 |
| **EPA + DHA** | **3.5 ± 0.03^a^** | **4.1 ± 0.08^b^** | **5.0 ± 0.01^c^** | **9.5 ± 0.06^d^** | **<0.0001** |
| **Σ PUFA^3^** | **31.3 ± 0.08^a^** | **31.4 ± 0.11^a^** | **31.9 ± 0.10^b^** | **33.7 ± 0.07^c^** | **<0.0001** |
| **Σ *n*-3** | **11.6 ± 0.03^a^** | **12.2 ± 0.09^b^** | **13.3 ± 0.07^c^** | **17.6 ± 0.08^d^** | **<0.0001** |
| **Σ *n*-6** | **19.6 ± 0.07^a^** | **19.2 ± 0.05^b^** | **18.6 ± 0.05^c^** | **15.8 ± 0.06^d^** | **<0.0001** |

^a,b,c,d^ Mean values within a row with unlike superscript letters were significantly different (*P* < 0.05; one-way ANOVA followed by Tukey’s honest significant difference test).

^1^Includes 15:0, 17:0, 20:0, 22:0, 24:0.

^2^Includes 14:1 *n*-5, 16:1 *n*-5, 16:1 *n*-9, 17:1 *n*-7, 18:1 *n*-11, 20:1 *n*-7, 20:1 *n*-11, 22:1 *n*-9, 24:1 *n*-9.

^3^Includes 16:2 *n*-3, 16:2 *n*-6, 16:3 *n*-4, 18:3 *n*-6, 18:4 *n*-3, 20:4 *n*-3.

MUFA, monounsaturated fatty acid; PUFA, polyunsaturated fatty acid; SFA, saturated fatty acid.

**Table S9. Total lipid content and fatty acid composition (% of total) in the middle intestine of Atlantic salmon fed different experimental diets. Data are shown as mean ± SEM (n = 3 cages).**

|  | **1.0% EPA + DHA** | **1.3% EPA + DHA** | **1.6% EPA + DHA** | **3.5% EPA + DHA** | ***P*-value (ANOVA)** |
| --- | --- | --- | --- | --- | --- |
| **Total fat** | **5.9 ± 0.95** | **5.0 ± 0.25** | **5.1 ± 0.38** | **4.9 ± 0.74** | **0.6707** |
| 14:0 | 0.8 ± 0.01^a^ | 0.9 ± 0.03^a^ | 1.1 ± 0.05^a^ | 1.6 ± 0.10^b^ | <0.0001 |
| 16:0 | 9.6 ± 0.38^a^ | 10.8 ± 0.24^ab^ | 11.4 ± 0.37^ab^ | 13.0 ± 0.98^b^ | 0.0183 |
| 18:0 | 3.7 ± 0.25 | 4.4 ± 0.19 | 4.6 ± 0.21 | 4.9 ± 0.45 | 0.1105 |
| **Σ SFA^1^** | **15.3 ± 0.70^a^** | **17.4 ± 0.37^ab^** | **18.5 ± 0.56^ab^** | **20.8 ± 1.60^b^** | **0.0191** |
| 16:1 *n*-7 | 0.9 ± 0.02^a^ | 0.9 ± 0.03^a^ | 1.2 ± 0.08^a^ | 1.7 ± 0.08^b^ | <0.0001 |
| 18:1 *n*-9 | 39.6 ± 1.20^a^ | 35.4 ± 0.78^ab^ | 33.0 ± 1.25^ab^ | 27.8 ± 3.05^b^ | 0.0106 |
| 18:1 *n*-7 | 2.6 ± 0.02^a^ | 2.6 ± 0.02^a^ | 2.6 ± 0.04^a^ | 2.8 ± 0.02^b^ | 0.0026 |
| 20:1 *n*-9 | 2.2 ± 0.07 | 2.2 ± 0.01 | 2.2 ± 0.07 | 2.4 ± 0.06 | 0.0893 |
| 22:1 *n*-7 | 0.7 ± 0.04 | 0.7 ± 0.02 | 0.6 ± 0.12 | 0.6 ± 0.02 | 0.3161 |
| 22:1 *n*-11 | 0.3 ± 0.01^a^ | 0.3 ± 0.01^a^ | 0.3 ± 0.03^a^ | 0.7 ± 0.02^b^ | <0.0001 |
| **Σ MUFA^2^** | **47.8 ± 1.19^a^** | **43.6 ± 0.87^ab^** | **41.2 ± 1.43^ab^** | **37.8 ± 3.13^b^** | **0.0291** |
| 18:2 *n*-6 | 15.7 ± 0.55^a^ | 14.0 ± 0.33^ab^ | 12.8 ± 0.44^bc^ | 11.0 ± 0.98^c^ | 0.0042 |
| 20:2 *n*-6 | 1.3 ± 0.04 | 1.5 ± 0.04 | 1.5 ± 0.07 | 1.3 ± 0.08 | 0.0511 |
| 20:3 *n*-6 | 0.7 ± 0.28 | 1.0 ± 0.07 | 0.9 ± 0.03 | 0.5 ± 0.07 | 0.1694 |
| 20:4 *n*-6 | 0.8 ± 0.10 | 1.0 ± 0.08 | 1.2 ± 0.07 | 1.5 ± 0.21 | 0.0268 |
| 18:3 *n*-3 | 5.5 ± 0.28^a^ | 4.8 ± 0.14^ab^ | 4.4 ± 0.20^ab^ | 3.6 ± 0.48^b^ | 0.0112 |
| 20:3 *n*-3 | 0.4 ± 0.02^ab^ | 0.4 ± 0.01^a^ | 0.4 ± 0.01^a^ | 0.3 ± 0.01^b^ | 0.0051 |
| 20:5 *n*-3 | 3.2 ± 0.22^a^ | 4.1 ± 0.13^a^ | 4.4 ± 0.18^ab^ | 6.1 ± 0.71^b^ | 0.0046 |
| 22:5 *n*-3 | 1.1 ± 0.09^a^ | 1.6 ± 0.12^ab^ | 1.7 ± 0.06^ab^ | 2.1 ± 0.26^b^ | 0.0144 |
| 22:6 *n*-3 | 6.4 ± 0.78^a^ | 9.1 ± 0.50^ab^ | 10.2 ± 0.63^ab^ | 13.0 ± 1.80^b^ | 0.0151 |
| **EPA + DHA** | **9.6 ± 1.00^a^** | **13.2 ± 0.62^ab^** | **14.6 ± 0.80^ab^** | **19.1 ± 2.51^b^** | **0.0109** |
| **Σ PUFA^3^** | **35.9 ± 0.22^a^** | **38.3 ± 0.55^ab^** | **38.2 ± 0.57^ab^** | **40.4 ± 1.55^b^** | **0.0422** |
| **Σ *n*-3** | **16.9 ± 0.77^a^** | **20.2 ± 0.61^ab^** | **21.4 ± 0.62^ab^** | **25.5 ± 2.30^b^** | **0.0099** |
| **Σ *n*-6** | **18.9 ± 0.71^a^** | **17.9 ± 0.18^a^** | **16.6 ± 0.39^ab^** | **14.6 ± 0.83^b^** | **0.0046** |

^a,b,c,d^ Mean values within a row with unlike superscript letters were significantly different (*P* < 0.05; one-way ANOVA followed by Tukey’s honest significant difference test).

^1^Includes 15:0, 17:0, 20:0, 22:0, 24:0.

^2^Includes 14:1 *n*-5, 16:1 *n*-5, 16:1 *n*-9, 17:1 *n*-7, 18:1 *n*-11, 20:1 *n*-7, 20:1 *n*-11, 22:1 *n*-9, 24:1 *n*-9.

^3^Includes 16:2 *n*-3, 16:2 *n*-6, 16:3 *n*-4, 18:3 *n*-6, 18:4 *n*-3, 20:4 *n*-3.

MUFA, monounsaturated fatty acid; PUFA, polyunsaturated fatty acid; SFA, saturated fatty acid.

**Table S10. Total lipid content and fatty acid composition (% of total) in the distal intestine of Atlantic salmon fed different experimental diets. Data are shown as mean ± SEM (n = 3 cages).**

|  | **1.0% EPA + DHA** | **1.3% EPA + DHA** | **1.6% EPA + DHA** | **3.5% EPA + DHA** | ***P*-value (ANOVA)** |
| --- | --- | --- | --- | --- | --- |
| **Total fat** | **3.9 ± 0.54** | **2.7 ± 0.22** | **3.8 ± 0.20** | **5.7 ± 1.25** | **0.0938** |
| 14:0 | 0.7 ± 0.03^a^ | 0.8 ± 0.06^ab^ | 1.0 ± 0.01^b^ | 2.1 ± 0.10^c^ | <0.0001 |
| 16:0 | 10.8 ± 0.34 | 12.0 ± 0.83 | 11.4 ± 0.42 | 11.8 ± 0.49 | 0.4529 |
| 18:0 | 4.4 ± 0.24 | 5.1 ± 0.45 | 4.3 ± 0.15 | 3.9 ± 0.25 | 0.1051 |
| **Σ SFA^1^** | **17.1 ± 0.53** | **19.1 ± 1.28** | **17.9 ± 0.49** | **19.0 ± 0.64** | **0.3073** |
| 16:1 *n*-7 | 0.7 ± 0.04^a^ | 0.8 ± 0.10^a^ | 1.0 ± 0.04^a^ | 2.3 ± 0.17^b^ | <0.0001 |
| 18:1 *n*-9 | 32.8 ± 1.49 | 28.1 ± 3.03 | 31.3 ± 1.20 | 29.2 ± 1.65 | 0.3870 |
| 18:1 *n*-7 | 2.6 ± 0.02^a^ | 2.5 ± 0.09^a^ | 2.7 ± 0.02^a^ | 3.0 ± 0.04^b^ | 0.0011 |
| 20:1 *n*-9 | 3.0 ± 0.11^ab^ | 3.2 ± 0.12^a^ | 3.0 ± 0.06^ab^ | 2.7 ± 0.08^b^ | 0.0229 |
| 22:1 *n*-7 | 0.8 ± 0.01 | 0.6 ± 0.10 | 0.7 ± 0.01 | 0.7 ± 0.02 | 0.1520 |
| 22:1 *n*-11 | 0.2 ± 0.02^a^ | 0.2 ± 0.05^a^ | 0.3 ± 0.04^a^ | 0.8 ± 0.06^b^ | <0.0001 |
| **Σ MUFA^2^** | **41.8 ± 1.61** | **37.3 ± 3.00** | **40.9 ± 1.26** | **40.8 ± 1.69** | **0.4517** |
| 18:2 *n*-6 | 12.3 ± 0.45 | 10.3 ± 1.20 | 11.2 ± 0.47 | 10.8 ± 0.71 | 0.3733 |
| 20:2 *n*-6 | 2.3 ± 0.14^a^ | 2.6 ± 0.20^a^ | 2.1 ± 0.07^ab^ | 1.5 ± 0.17^b^ | 0.0092 |
| 20:3 *n*-6 | 1.1 ± 0.04^a^ | 1.0 ± 0.12^ab^ | 0.7 ± 0.03^b^ | 0.3 ± 0.01^c^ | <0.0001 |
| 20:4 *n*-6 | 1.3 ± 0.13 | 1.5 ± 0.18 | 1.3 ± 0.10 | 1.1 ± 0.14 | 0.3322 |
| 18:3 *n*-3 | 4.4 ± 0.19 | 3.7 ± 0.45 | 4.2 ± 0.19 | 4.1 ± 0.30 | 0.4934 |
| 20:3 *n*-3 | 0.6 ± 0.02^a^ | 0.6 ± 0.02^a^ | 0.6 ± 0.01^a^ | 0.5 ± 0.04^b^ | 0.0072 |
| 20:5 *n*-3 | 4.7 ± 0.32 | 5.7 ± 0.69 | 5.1 ± 0.23 | 5.7 ± 0.22 | 0.2939 |
| 22:5 *n*-3 | 2.1 ± 0.11 | 2.7 ± 0.32 | 2.4 ± 0.10 | 2.7 ± 0.24 | 0.2799 |
| 22:6 *n*-3 | 10.4 ± 0.96 | 13.8 ± 1.87 | 11.9 ± 0.81 | 11.5 ± 1.48 | 0.3971 |
| **EPA + DHA** | **15.1 ± 1.26** | **19.5 ± 2.56** | **17.0 ± 1.05** | **17.1 ± 1.68** | **0.4039** |
| **Σ PUFA^3^** | **40.0 ± 1.03** | **42.7 ± 1.78** | **40.3 ± 0.68** | **39.1 ± 1.12** | **0.2560** |
| **Σ *n*-3** | **22.4 ± 1.24** | **26.8 ± 2.43** | **24.5 ± 0.95** | **24.8 ± 1.57** | **0.3649** |
| **Σ *n*-6** | **17.2 ± 0.29^a^** | **15.5 ± 0.68^ab^** | **15.6 ± 0.27^ab^** | **14.1 ± 0.52^b^** | **0.0112** |

^a,b,c^ Mean values within a row with unlike superscript letters were significantly different (*P* < 0.05; one-way ANOVA followed by Tukey’s honest significant difference test).

^1^Includes 15:0, 17:0, 20:0, 22:0, 24:0.

^2^Includes 14:1 *n*-5, 16:1 *n*-5, 16:1 *n*-9, 17:1 *n*-7, 18:1 *n*-11, 20:1 *n*-7, 20:1 *n*-11, 22:1 *n*-9, 24:1 *n*-9.

^3^Includes 16:2 *n*-3, 16:2 *n*-6, 16:3 *n*-4, 18:3 *n*-6, 18:4 *n*-3, 20:4 *n*-3.

MUFA, monounsaturated fatty acid; PUFA, polyunsaturated fatty acid; SFA, saturated fatty acid.

**Table S11. Visual evaluation of external and internal welfare indicators. External and internal welfare indexes are also included.**

|  | **Dietary EPA+DHA levels (%)** | | | | | | | | | | | | | |  | |  |
| --- | --- | --- | --- | --- | --- | --- | --- | --- | --- | --- | --- | --- | --- | --- | --- | --- | --- |
|  | **1** | |  | | **1.3** | |  | | **1.6** | |  | | **3.5** | |  | |  |
|  | **Mean** | **SEM** | | **Mean** | | **SEM** | | **Mean** | | **SEM** | | **Mean** | | **SEM** | ***P*-value (ANOVA)** |  |  |
| **External indicators** | |  |  | |  | |  | |  | |  | |  | |  |  |  |
| **External welfare index** | | 2.20 | 0.50 | | 1.49 | | 0.12 | | 1.61 | | 0.19 | | 1.48 | | 0.11 | NS |  |
| Eyes | | 0.46 | 0.07 | | 0.64 | | 0.14 | | 0.47 | | 0.08 | | 0.33 | | 0.07 | NS | |
| Skin | | 0.64 | 0.06 | | 0.69 | | 0.10 | | 0.52 | | 0.08 | | 0.85 | | 0.28 | NS | |
| Snout | | 1.22 | 0.02 | | 1.37 | | 0.05 | | 1.12 | | 0.05 | | 1.17 | | 0.07 | NS | |
| Dorsal fin | | 1.36 | 0.15 | | 1.04 | | 0.12 | | 1.02 | | 0.16 | | 0.82 | | 0.08 | NS | |
| Caudal fin | | 1.16 | 0.25 | | 1.13 | | 0.04 | | 1.04 | | 0.04 | | 0.98 | | 0.08 | NS | |
| Pectoral fin | | 0.93 | 0.31 | | 1.22 | | 0.10 | | 1.00 | | 0.24 | | 0.69 | | 0.12 | NS | |
| **Internal indicators** | |  |  | |  | |  | |  | |  | |  | |  |  | |
| **Internal welfare index** | | 4,59^a^ | 0,07 | | 4,45^a^ | | 0,15 | | 3,72^c^ | | 0,09 | | 2,68^d^ | | 0,14 | <0.0001 | |
| Melanin adipose tissue | | 1.23 | 0.13 | | 1.25 | | 0.02 | | 1.11 | | 0.08 | | 1.01 | | 0.01 | NS | |
| Melanin heart | | 1.11^a^ | 0.04 | | 1.00^b^ | | 0.00 | | 1.00^b^ | | 0.00 | | 1.00^b^ | | 0.00 | 0.018 | |
| Heart shape | | 1.50 | 0.10 | | 1.44 | | 0.14 | | 1.36 | | 0.12 | | 1.38 | | 0.14 | NS | |
| Stomach inflammation | | 1.02 | 0.02 | | 1.00 | | 0.00 | | 1.00 | | 0.00 | | 1.03 | | 0.03 | NS | |
| Intestine inflammation | | 1.26 | 0.07 | | 1.20 | | 0.05 | | 1.13 | | 0.07 | | 1.03 | | 0.03 | NS | |
| Liver fat | | 1.89^a^ | 0.05 | | 1.98^a^ | | 0.32 | | 1.62^ab^ | | 0.11 | | 0.65^b^ | | 0.31 | 0.013 | |

Data are shown as mean ± SEM (*n* = 3 cages). Different letters within each row indicate significant differences between values determined using a one-way ANOVA followed by Tukey's post hoc test.

**Supplementary figures**


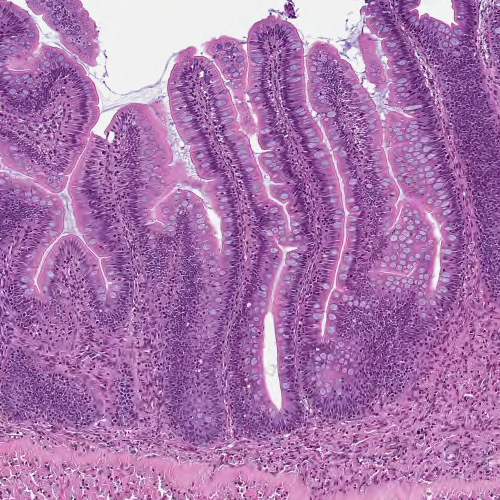

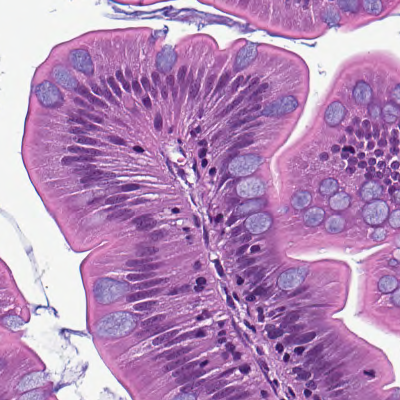

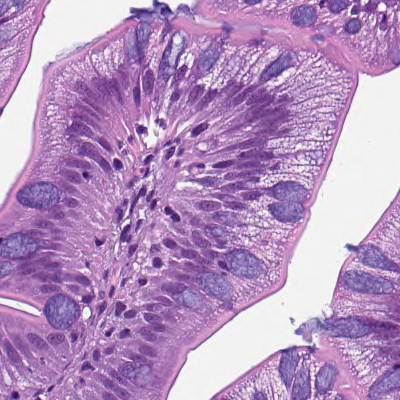


**C**

**B**

**A**

**Figure S1.** Middle intestine. (**A**) Normal folding of middle intestine mucosa, (**B**) detail of mucosal lining with non-vacuolized cytoplasm of enterocytes, and (**C**) detail of middle intestine mucosa with signs of excess vacuolization of enterocytes.


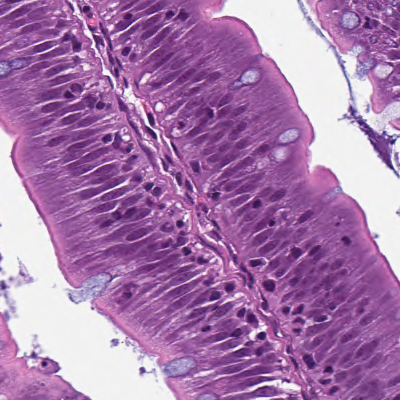

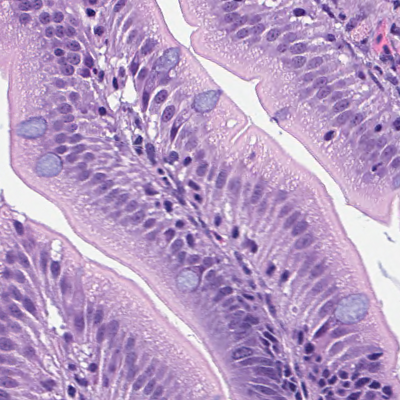

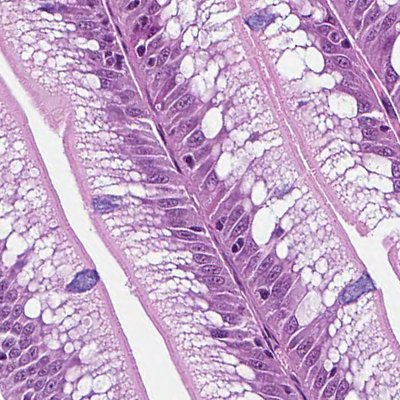


**C**

**B**

**A**

**Figure S2.** Detail of distal intestine mucosa. (**A**) Section of intestinal fold with supranuclear cytoplasm of enterocytes devoid of vacuolization, (**B**) intestinal mucosa with foamy cytoplasm, and (**C**) normal vacuolization of distal intestinal enterocytes.

| ***n*** | **Fish with fusions (%)** | **Average size of fusion (mm)** | **Fish with cross-stitch lesions (%)** |
| --- | --- | --- | --- |
| 5 | 16 | 9 | 4 |
| 22 | 18 | 4 | 5 |
| 24 | 21 | 4 | 0 |
| 24 | 17 | 4 | 4 |


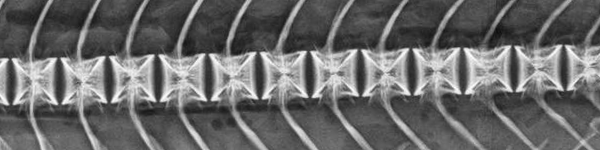


**A**


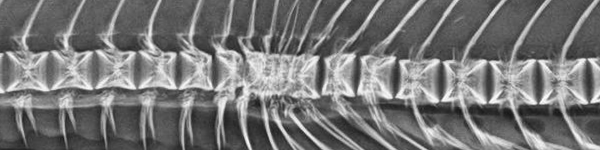


**B**


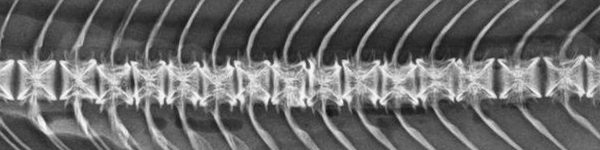


**C**

**Figure S3.** Detail of vertebral lesions (radiography). (**A**) Section of normal vertebral column, (**B**) fusion affecting a total of 11 vertebrae (circled), and (**C**) section of vertebral column with cross-stitch vertebrae (dotted line).
